# Supramolecularly engineered phospholipids constructed by nucleobase molecular recognition: upgraded generation of phospholipids for drug delivery†
†Electronic supplementary information (ESI) available: Synthesis detail and characterization of supramolecular nucleoside phospholipids; fabrication of supramolecular liposomes and conventional liposomes, including fabrication of DOX-loaded supramolecular liposomes and conventional liposomes; details of *in vitro* experiments, including *in vitro* cytotoxicity studies, intracellular drug release, examination of cell viability of MCF-7 cells, apoptosis analyses and western blotting analysis; details of *in vivo* experiments, including pharmacokinetic studies, *in vivo* biodistribution and tumor targeting capability in tumor-bearing mice and *in vivo* antitumor efficacy. See DOI: 10.1039/c5sc01188d


**DOI:** 10.1039/c5sc01188d

**Published:** 2015-05-12

**Authors:** Dali Wang, Chunlai Tu, Yue Su, Chuan Zhang, Udo Greiser, Xinyuan Zhu, Deyue Yan, Wenxin Wang

**Affiliations:** a School of Chemistry and Chemical Engineering , State Key Laboratory of Metal Matrix Composites , Shanghai Jiao Tong University , 800 Dongchuan Road , Shanghai 200240 , People's Republic of China . Email: xyzhu@sjtu.edu.cn ; Fax: +86-21-54741297 ; Tel: +86-21-34203400; b Charles Institute of Dermatology , School of Medicine and Medical Science , University College Dublin , Belfield , Dublin 4 , Ireland . Email: wenxin.wang@ucd.ie

## Abstract

Despite of great advances of phospholipids and liposomes in clinical therapy, very limited success has been achieved in the preparation of smart phospholipids and controlled-release liposomes for *in vivo* drug delivery and clinical trials. Here we report a supramolecular approach to synthesize novel supramolecularly engineered phospholipids based on complementary hydrogen bonding of nucleosides, which greatly reduces the need of tedious chemical synthesis, including reducing the strict requirements for multistep chemical reactions, and the purification of the intermediates and the amount of waste generated relative more traditional approaches. These upgraded phospholipids self-assemble into liposome-like bilayer structures in aqueous solution, exhibiting fast stimuli-responsive ability due to the hydrogen bonding connection. *In vitro* and *in vivo* evaluations show the resulted supramolecular liposomes from nucleoside phospholipids could effectively transport drug into tumor tissue, rapidly enter tumor cells, and controllably release their payload in response to an intracellular acidic environment, thus resulting in a much higher antitumor activity than conventional liposomes. The present supramolecularly engineered phospholipids represent an important evolution in comparison to conventional covalent-bonded phospholipid systems.

## Abstract 2

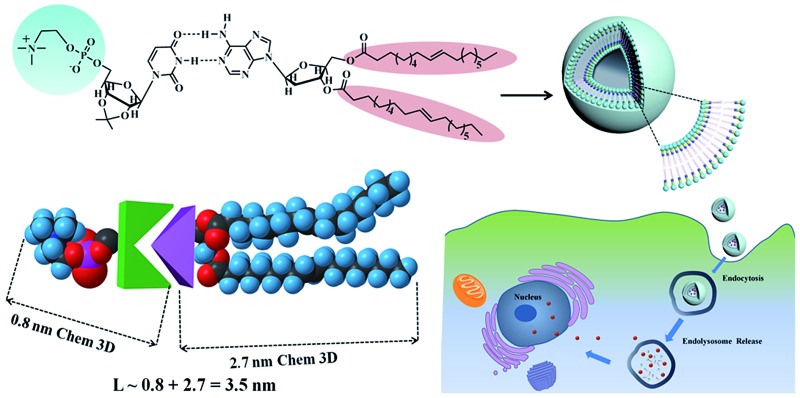
Supramolecularly engineered phospholipids and liposomes based on complementary hydrogen bonding of nucleosides have been developed.

## Introduction

Since their discovery several decades ago, phospholipids and liposomes have become one of the increasingly significant topics in chemistry and biology because of their importance in biological systems.^1^–^6^ Conventional glycerol-based phospholipids such as phosphatidylcholine (PC) and phosphatidylethanolamine (PE) can self-assemble into three-dimensional hollow spheres with self-closed structures in water known as liposomes.^7^,^8^ Owing to their intrinsic biocompatibility and unique self-assembly behavior, phospholipids and liposomes have been widely applied in the fields of biotechnology, drug carriers, gene delivery, contrast agents, as well as the surface modification of biomaterials.^9^–^13^ So far, several liposome-based drug delivery systems have been approved by the Food and Drug Administration (FDA) and numerous liposome-encapsulated agents have been used in clinical trials.^14^–^17^ However, the encapsulated drugs in conventional liposomes could not be released efficiently at the target sites in a controlled fashion, which limits their clinical application greatly.^10^,^18^ To address this challenge, various stimuli-responsive liposomes including temperature-, pH-, redox- and enzyme-responsive ones have been developed to improve the drug bioavailability.^19^–^24^ Among these stimuli, the pH trigger is the most extensively studied one because mildly acidic environments are encountered in tumor sites, as well as in intracellular compartments such as endosomes and lysosomes of cells.^25^,^26^ A variety of pH-sensitive phospholipids have been developed as smart carriers containing different pH-sensitive linkers, such as acetal, ketal, vinyl ether and ortho ester.^27^–^31^ However, the chemical bonds in the aforementioned covalent phospholipids usually cannot promptly respond to the mildly acidic pH condition, which limits the fast release of the loaded cargos from liposomes for therapeutic purposes. Moreover, these pH-sensitive and covalently bonded phospholipids are not easy to be prepared and generally require tedious synthesis work, which prevents them from practical pharmaceutical development. To date, very limited progress has been achieved on the responsive phospholipids and liposomes with high sensitivity and ease of chemical synthesis for *in vivo* drug delivery and clinical trials.^14^,^31^

Compared to conventional phospholipids containing a covalent bond between the phospholipid head group and tail, herein we proposed and constructed a new type of phospholipid using non-covalent molecular recognition to link the head group and tail together. Inspired by biological systems in which multiple hydrogen bonding interactions occur in the adenine–uracil (A–U), adenine–thymine (A–T) and guanine–cytosine (G–C) base pairs in DNA and RNA,^32^–^34^ in our new strategy, hydrophilic head and hydrophobic tails of phospholipids are engineered and linked together through strong multiple hydrogen bonding interactions of nucleobases. As a proof-of-concept, we synthesized supramolecular nucleoside phospholipids using uridine-functionalized PC or PE as hydrophilic head and adenosine-functionalized myristic acid or oleic acid as hydrophobic tails. Through the molecular recognition between adenosine (A) and uridine (U), these components formed four different kinds of supramolecular nucleoside phospholipids *via* a simple mixing procedure. The obtained supramolecular nucleoside phospholipids could further self-assemble into liposome-like bilayer nano-vesicles in aqueous solution because of their amphiphilic property.^35^–^39^ It is well known that complementary multiple hydrogen bonding interactions are moderately strong, highly directional and sensitive to acidic pH,^40^–^43^ thus the liposomes prepared from these new supramolecular phospholipids exhibited high sensitivity to acidic stimuli. With these hallmark properties, we demonstrated that the doxorubicin-loaded (DOX-loaded) supramolecular liposomes exhibited higher anticancer efficacy over conventional liposome counterparts constructed by 1,2-dioleoyl-*sn*-glycero-3-phosphocholine (DOPC) in both *in vitro* and *in vivo*.

## Results and discussion

### Synthesis and characterization of supramolecular nucleoside based phospholipids

**Fig. 1:**
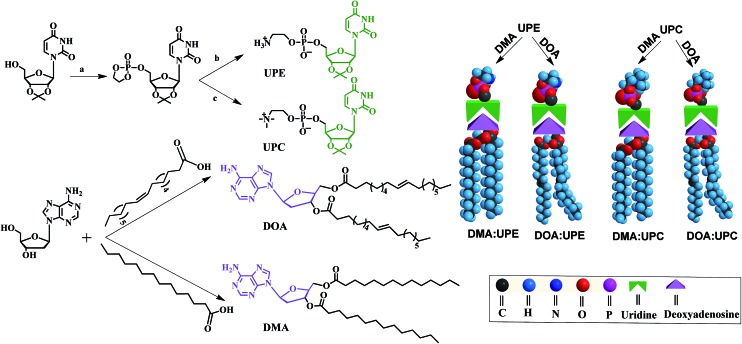
Synthetic route, chemical structures of nucleoside phospholipids and schematic representation for the formation of supramolecular phospholipids. *Reagents and conditions*: (a) chlorooxodioxaphospholane, TEA, THF, 0 °C, 15 h; (b) trimethylamine, acetonitrile, THF, 60 °C, 24 h. (c) Ammonia, acetonitrile, THF, 65 °C, 48 h. UPE and UPC are uridine-functionalized PE and PC as hydrophilic phospholipid head, respectively. DMA and DOA are adenosine-functionalized myristic acid and oleic acid as hydrophobic tails, respectively. Through the molecular recognition between adenosine and uridine, these two components form four different types of supramolecular nucleoside phospholipids (DMA : UPE, DOA : UPE, DMA : UPC and DOA : UPC) by mixing a uridine-terminated head and an adenosine-terminated tail.

Phospholipids are the major lipid component in cell membranes. Among them, PC and PE are the most common phospholipids in biological membranes. Generally, these conventional phospholipid molecules consist of a hydrophilic polar head group and a pair of nonpolar fatty acid tails, connected *via* a glycerol linkage. In our work, we designed and synthesized two uridine-functionalized hydrophilic phospholipid heads (uridine acetonide phosphatidylethanolamine, UPE; uridine acetonide phosphatidylcholine, UPC) and two adenosine-functionalized hydrophobic tails (3′,5′-dimyristoyladenosine, DMA; 3′,5′-dioleoyladenosine, DOA), the structures of which are shown in Fig. 1. The synthesis of these nucleoside-functionalized components is shown in Fig. 1 and S1 of the ESI.† Briefly, in the presence of triethylamine (TEA), the uridine acetonide reacted with an excess of chlorooxodioxaphospholane in tetrahydrofuran (THF) at 0 °C to yield uridine-oxo-dioxaphospholane phosphate. This phosphate was transferred to a pressure tube and heated for 24 h with TEA in acetonitrile to give UPC. UPE was synthesized using two different procedures. In the first procedure, reaction with uridine-oxo-dioxaphospholane phosphate was performed based on a similar method that was described for the synthesis of UPC (Fig. 1). In the second procedure, the uridine acetonide derivative **1** was reacted with phosphorus oxychloride in THF in the presence of TEA at room temperature to obtain the phosphate nucleoside derivative **2**. Compound **2** was treated with ethanolamine and TEA in THF to give **3**. Then the UPE was obtained by hydrolysis of **3** using acetic acid (Fig. S1†). In the next step, the two fatty chains (myristic acid or oleic acid) were coupled to two hydroxyl groups of 2′-deoxyadenosine by esterification reaction in the presence of 1-ethyl-3-(3-dimethyllaminopropyl)carbodiimide hydrochloride and 4-dimethylaminopyridine to synthesize DMA and DOA as shown in Fig. 1. The products were purified by size exclusion chromatography (dichloromethane–methanol, 20 : 1). The structures of the resulting nucleoside-functionalized components were confirmed by nuclear magnetic resonance (NMR) and high-resolution mass spectroscopy (HRMS) (ESI Fig. S2–S13†).

**Fig. 2:**
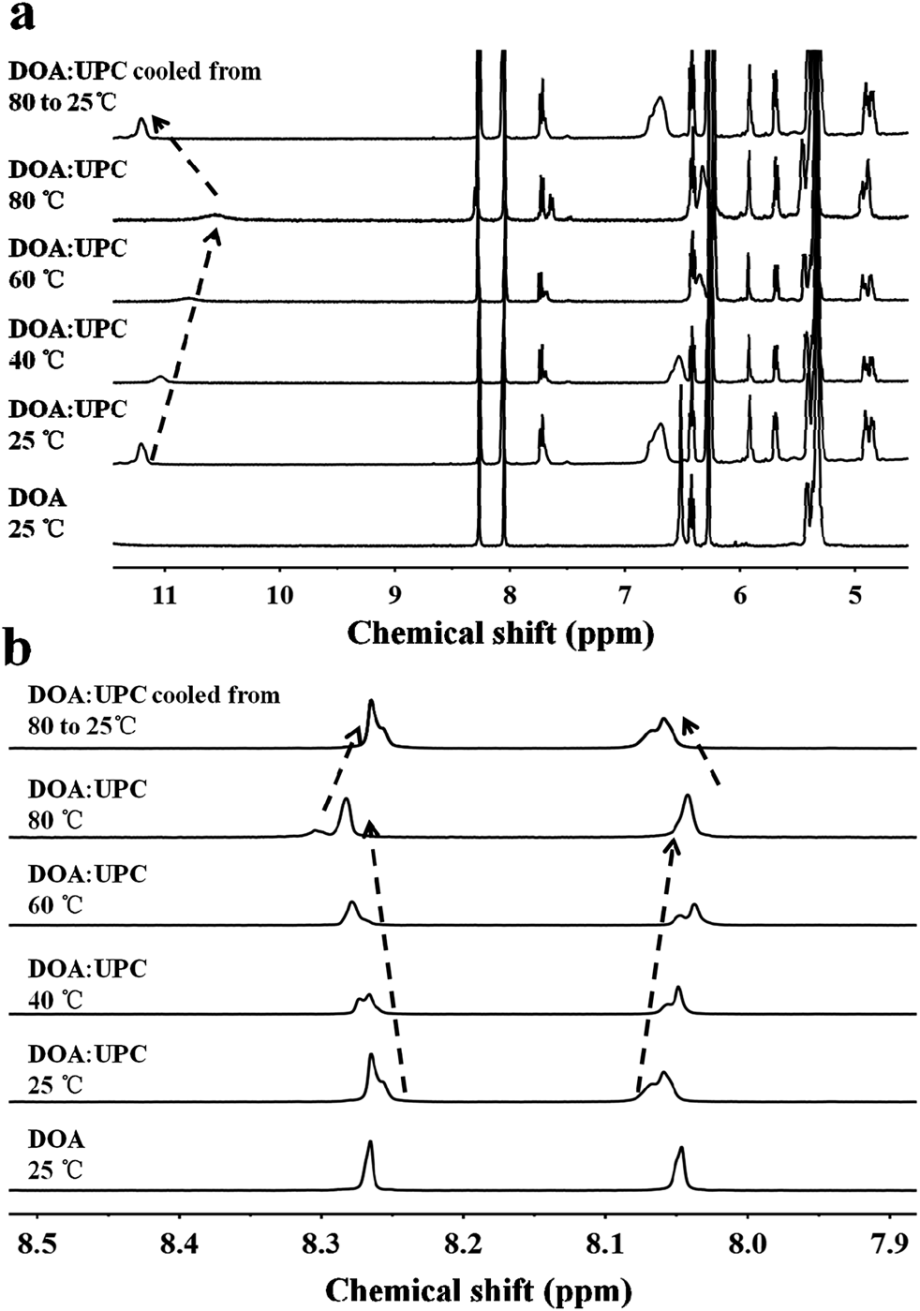
Variable-temperature ^1^H NMR spectra of DOA and DOA : UPC. (a) ^1^H NMR spectrum of DOA at 25 °C (bottom) and ^1^H NMR spectra of DOA : UPC at 25, 40, 60, 80 °C and cooled from 80 to 25 °C in the 4.5–11.5 ppm region. The arrow represents the changing trend of uracil NH chemical shift. (b) ^1^H NMR spectrum of DOA at 25 °C (bottom) and ^1^H NMR spectra of DOA : UPC at 25, 40, 60, 80 and cooled from 80 to 25 °C in the 7.9–8.5 ppm region. The arrows represent the changing trend of adenine CH chemical shifts. The mixing ratio (DOA : UPC) was 1 : 1. The sample was allowed to equilibrate for 5 min at each temperature (1,1,2,2-tetrachloroethane-*d*_2_–dimethyl sulfoxide-*d*_6_, 4 : 1).

Due to the molecular recognition between the adenosine and uridine, the supramolecular nucleoside phospholipids were formed by mixing a uridine-functionalized hydrophilic head and an adenosine-functionalized hydrophobic tail. Fig. 1 depicts the formation of four types of supramolecular nucleoside phospholipids (DMA : UPE, DOA : UPE, DMA : UPC and DOA : UPC). The formation of complementary multiple hydrogen bonds between the supramolecular phospholipid head and the tail was analyzed by variable-temperature ^1^H NMR spectroscopy after blending the two compounds with an equivalent mole of adenine to uracil units in 1,1,2,2-tetrachloroethane-*d*_2_ and dimethylsulfoxide-*d*_6_ (4 : 1). The ^1^H NMR patterns of the supramolecular phospholipids were compared with the precursor of adenosine-functionalized fatty acid tails. Fig. 2a shows the temperature dependence of the NH proton chemical shift in the DOA and UPC complexes. It is found that the chemical shift of the NH resonance moves up-field systematically from 11.2 to 10.6 ppm when the temperature increases from 25 to 80 °C. The gradual decrease in the NH resonance with the increase of temperature can be attributed to the dissociation of the complementary hydrogen bonds.^44^ However, as soon as the sample is cooled from 80 to 25 °C, the NH resonance returns to its original position at 11.2 ppm. Fig. 2b shows that the peak shapes of adenine CH from DOA : UPC change apparently compared with that from DOA, and the chemical shifts of CH move with the increase of temperature. These data indicate the formation of supramolecular nucleoside phospholipids based on the complementary hydrogen bonds between DOA and UPC. Similarly, the hydrogen-bonding interactions of DMA : UPE, DOA : UPE and DMA : UPC are confirmed by variable-temperature ^1^H NMR (ESI Fig. S14–S16†).

### Supramolecular assemblies of nucleoside phospholipids

**Fig. 3:**
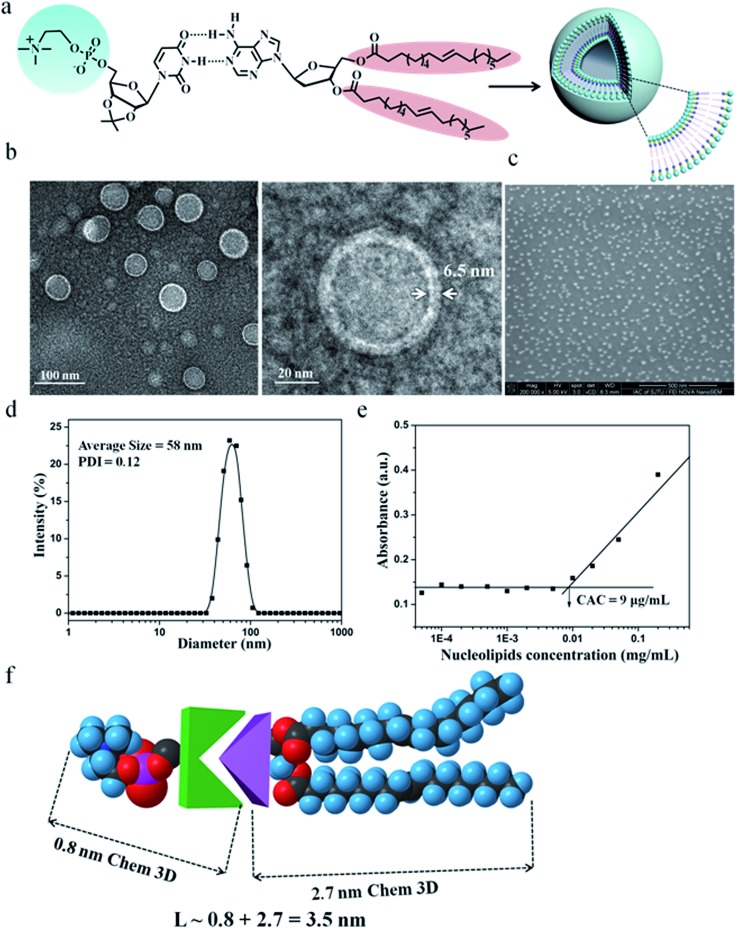
Characterization of molecular self-assembly of supramolecular nucleoside phospholipids DOA : UPC. (a) Schematic representation of a supramolecular liposome self-assembled from the DOA : UPC nucleoside phospholipids. Supramolecular nucleoside phospholipids self-assemble into liposome-like bilayer structures in aqueous solution. (b) Representative TEM images of negatively stained supramolecular DOA : UPC liposomes. The liposome wall thickness is about 6.5 nm. (c) Representative SEM image of supramolecular DOA : UPC liposomes (scale bars are 500 nm). (d) DLS profile for the supramolecular liposomes. (e) Relationship of the absorbance and the concentration of DOA : UPC in aqueous solutions. (*λ* = 313 nm, 25 °C). (f) Estimation of the length of an extended DOA : UPC molecule according to the Chem3D results.

Owing to the presence of the hydrophobic and hydrophilic domains, the supramolecular nucleoside phospholipids self-assembled into nano-scale particles in aqueous solution (Fig. 3 and ESI Fig. S17†). Fig. 3a shows the amphipathic structure of DOA : UPC and their self-assembly behavior. The morphology of the DOA : UPC particles was observed by transmission electron microscopy (TEM) and scanning electron microscopy (SEM). Before being analyzed by TEM, the particles were disposed by the negative-staining technique (2% potassium phosphotungstate at pH 6.8–7.4).^45^ The resulting micrographs are displayed in Fig. 3b. The particles show a clear contrast between the particle skin and the inner pool, suggesting a vesicular structure.^46^,^47^ The vesicle wall thickness is almost uniform, about 6.5 nm through the statistical analysis of 30 vesicles from the TEM images. Considering that the extended length of DOA : UPC calculated with Chem3D is around 3.5 nm (Fig. 3f), the vesicles may possess a bilayer structure with two hydrophilic PC shell layers and two hydrophobic alkyl chain core layers (Fig. 3a). Therefore, we rationalize that supramolecular nucleoside phospholipids have successfully self-assembled into spherical self-closed liposome-like structures with diameters ranging from 50 to 60 nm. The SEM images of the supramolecular aggregates show uniform spherical particles, and the average diameter around 56 nm is similar to that observed by TEM (Fig. 3c). The size distribution of the DOA : UPC vesicles was also determined by dynamic light scattering (DLS), and Fig. 3d gives a monomodal size distribution with the *Z*-average diameter of 58 nm with a polydispersity index (PDI) of 0.12, which is in accordance with the results from TEM and SEM measurements. As a control, the assemblies from the pure DOA were also prepared and observed by TEM under the same condition as DOA : UPC. It is found that the pure DOA self-assembles into aggregates with diameters about 40 nm (ESI Fig. S17†).

In order to investigate the self-assembly ability of DOA : UPC in water, the critical aggregation concentration (CAC) measurements were performed by using 1,6-diphenyl-1,3,5-hexatriene (DPH) as a hydrophobic probe. As shown in Fig. 3e, this supramolecular phospholipid has a relatively low CAC (9 μg mL^–1^), indicating the high stability of DOA : UPC liposomes. The self-assembly behaviors of supramolecular nucleoside phospholipids DMA : UPE, DOA : UPE and DMA : UPC were also studied by TEM and DLS measurements. Similarly, TEM images of negatively stained nanoparticles demonstrate that they self-assemble into liposome-like bilayer structures with diameters ranging from 30 to 50 nm (ESI Fig. S18†). The DLS data show that the diameters of these supramolecular liposomes increase while increasing the hydrophobicity of the lipid tails (Table 1).

**Table 1:** Properties of liposomes formed from supramolecular nucleoside phospholipids^*b*^

Chemical structure	Diameter^*a*^ (nm)	DLC (%)	DLE (%)
DMA : UPE	42.4 ± 2.4	3.8	19
DMA : UPC	45.2 ± 1.9	4.2	21
DOA : UPE	54.3 ± 1.7	5.2	26
DOA : UPC	58.0 ± 2.5	6.4	32

^*a*^Diameter of supramolecular liposomes was determined by dynamic light scattering.

^*b*^Abbreviations: DLC, drug loading content; DLE, drug loading efficiency. All the measurements were performed in triplicate.

**Fig. 4:**
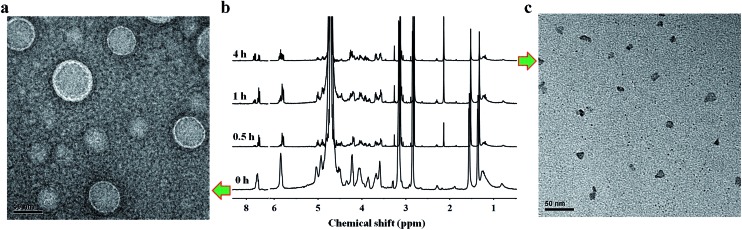
Acid-triggered destabilization of supramolecular DOA : UPC liposomes. (a) Representative TEM image of negatively stained supramolecular DOA : UPC liposomes at pH 7.4 (scale bars are 50 nm). (b) ^1^H NMR spectra of supramolecular DOA : UPC liposome suspensions before and after incubation at pH 5.0 in D_2_O–HCl (25 °C) for different periods of time. From bottom to top: supramolecular liposome suspensions were incubated at pH 7.4 in D_2_O and at pH 5.0 in D_2_O–HCl for 0.5, 1 and 4 h, respectively. The spectrum of the suspension at pH 7.4 is characterized by broad and featureless peaks. In contrast, the spectra after exposure to acidic stimuli are characterized by the sharpening and shifting of the peaks. (c) Representative TEM image of supramolecular DOA : UPC liposomes after incubation at pH 5.0 buffer for about 4 h (scale bars are 50 nm).

The disruption of hydrogen-bonding interactions between the hydrophilic phospholipid head and hydrophobic tails made nucleoside liposomes unstable at an acidic condition, endowing them with pH-responsive capability. To evaluate this emerging property, we treated the supramolecular DOA : UPC liposomes with acetate buffer (pH = 5.0) for 4 h and observed the morphology of supramolecular liposomes by TEM. As shown in Fig. 4c, exposure to acidic condition (pH 5.0) induces significant ripping or crumpling of the structures, as well as particle shrinkage. However, almost all nanoparticles are spherical and intact under neutral (pH 7.4) condition (Fig. 4a). Then we used ^1^H NMR spectroscopy of the supramolecular DOA : UPC liposome suspensions before and after exposure to acidic stimuli (pH 5.0) at different time intervals to characterize the nature of the observed morphological changes at the molecular level. The spectra in neutral aqueous buffer condition (pH 7.4, Fig. 4b, bottom) and after exposure to acidic medium (pH 5.0, Fig. 4b, top) demonstrate changes consistent with alteration in the environment of the nucleosides of supramolecular phospholipids, garnering insight from the width of the ^1^H NMR nucleoside peaks. The broad and featureless bands corresponding to the methine protons (7.8 ppm and 5.9 ppm) of nucleobases become sharp rapidly on exposure to acidic condition (Fig. 4b, bottom *vs*. Fig. 4b, top). When present in liposomal aggregates, the nucleobases exist in an almost water-free environment, so that the transverse relaxation time of the nucleobase protons is very short and consequently the resonances become very broad (Fig. 4b, bottom). The protonation of the nucleobase nitrogen atoms makes the liposomes disaggregate gradually, so that more water is now present in this domain, consequently increasing the transverse relaxation time and thus sharpening the signals (Fig. 4b, top). Both TEM and NMR results suggest that the supramolecular liposomes self-assembled from the DOA : UPC nucleoside phospholipids possess a rapid pH-sensitive dissolution property which may be exploited to engineer drug delivery vehicles that can release their payloads under mild acidic conditions in a controlled manner.

To evaluate the potential use of supramolecular liposomes based on nucleobase recognition as carriers to deliver therapeutic agents, doxorubicin (DOX) was selected as a model anticancer drug to determine their drug loading and release properties. DOX was loaded into the cavity of the supramolecular liposomes, similar to loading drugs into conventional liposomes.^48^ When the theoretical drug loading content (DLC) was set at 20 wt%,^12^ the drug loading contents of DMA : UPE, DMA : UPC, DOA : UPE and DOA : UPC were 3.8, 4.2, 5.2 and 6.4%, respectively, corresponding to drug loading efficiencies (DLE) of 19, 21, 26 and 32% (Table 1). Among them, the supramolecular DOA : UPC liposomes possess the highest drug load ability and efficiency because of its largest vesicle size.

**Fig. 5:**
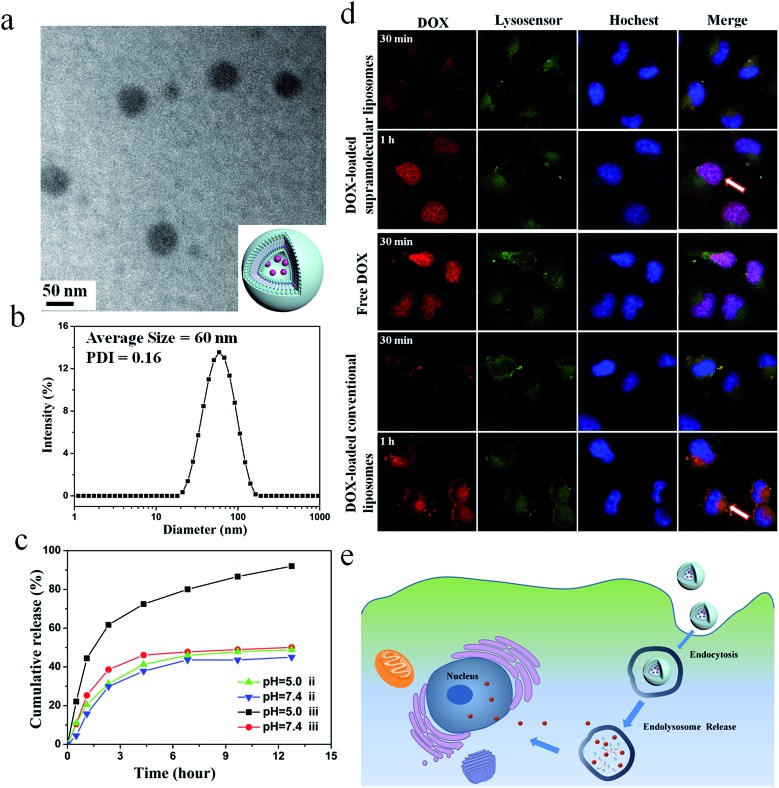
Characterization of DOX-loaded supramolecular liposomes self-assembled from nucleoside phospholipids DOA : UPC. (a) Representative TEM image of supramolecular liposomes with DOX (inset: schematic representation of a DOX-loaded supramolecular liposome). (b) DLS curve of DOX-loaded supramolecular liposomes with a concentration of 0.5 mg mL^–1^. (c) *In vitro* drug release kinetics from DOX-loaded conventional liposomes (ii) and supramolecular liposomes (iii) at different pH values (7.4 and 5.0) at 37 °C. (d and e) Cellular uptake of supramolecular liposomes from DOA : UPC and intracellular dug release. (d) Representative CLSM images of MCF-7 cells incubated with DOX-loaded supramolecular liposomes, DOX-loaded conventional liposomes and free DOX for 30 min and 1 h, respectively. Scale bar: 10 μm. (e) Schematic representation for proposed mechanism of cellular uptake of supramolecular liposomes and intracellular drug release.

The size of the particles is an important parameter that prevents renal clearance (typically <20 nm), avoids uptake by the liver and spleen (particles >150 nm), and enhances accumulation in the tumor (particles between 50–150 nm).^49^–^51^ Considering the high drug encapsulation and appropriate size, the supramolecular DOA : UPC liposomes were more suitable for intracellular drug delivery compared with the other three liposome types and thus the following study only focused on the DOA : UPC liposomes. Here, the conventional liposomes with a comparable size (about 55 nm) formed from DOPC were used as a control (ESI Fig. S19†). As depicted in Fig. 5a, DOX-loaded DOA : UPC vesicles are approximately 55 nm and have a dark core. Thus, loading of DOX into the particles further confirmed their vesicular structure. The DLS measurements demonstrate DOX-loaded DOA : UPC vesicles have a monomodal size distribution with the *Z*-average diameter of 60 nm and a PDI of 0.16 (Fig. 5b).

### Enhanced *in vitro* drug release from supramolecular liposomes at acidic pH

The *in vitro* release behaviors of DOX-loaded supramolecular liposomes and conventional liposomes were investigated under a simulated physiological condition (PBS, pH 7.4) and in an acidic environment (acetate buffer, pH 5.0) at 37 °C to assess the feasibility of using supramolecular liposomes as an anticancer drug delivery carrier. The drug release profiles of DOX are shown in Fig. 5c. It is found that DOX-loaded supramolecular liposomes present a relatively rapid release compared to DOX-loaded conventional liposomes. For conventional liposomes, the release rate of DOX at pH 5.0 is comparable to that at pH 7.4. However, the release rate of DOX from the supramolecular liposomes at pH 5.0 is much faster than that at pH 7.4, which indicates that pH of the medium has a strong effect on the DOX release from the supramolecular structure. At pH 7.4, beyond the initial burst release, the release rate of DOX from the supramolecular liposomes is relatively low with less than 50% of the released DOX in 12 h. At pH 5.0, the DOX release is apparently accelerated to approximately 75% of the released drug within 5 h. The fast release of the DOX-loaded supramolecular liposomes in an acidic environment is likely due to the protonation of the amino group of nucleobase and disaggregation of supramolecular liposomes at an acidic condition.

### Tracking of DOX-loaded supramolecular liposomes in MCF-7 cells

The cellular uptake and intracellular drug release of a drug carrier play important roles in successful drug delivery. Utilizing flow cytometry-based analysis, the cellular adhesion of supramolecular liposomes was estimated by measuring the intracellular fluorescence intensity. Fig. S20† shows the histograms of cell-associated DOX fluorescence intensity for MCF-7 cells (a human breast adenocarcinoma cell line) incubated with DOX-loaded supramolecular liposomes at the predetermined time intervals. Here, MCF-7 cells without any treatment were set as a control. The histogram of cells shifts to the direction of high fluorescence intensity with increasing time. The fast enhancement of fluorescence signals indicates high cellular adhesion of DOX-loaded supramolecular liposomes by MCF-7 cells, which facilitates the occurrence of cellular uptake.

The ability of DOX-loaded supramolecular liposomes to enter cancer cells was observed by confocal laser scanning microscopy (CLSM). As shown in Fig. 5d, DOX-loaded supramolecular liposomes are quickly internalized and mostly localized in the lysosomes (yellow spots in 30 min). Remarkably, strong DOX fluorescence is observed in the nuclei (pink spots in 1 h) after 1 h of incubation, which can be ascribed to the released DOX from internalized DOX-loaded supramolecular liposomes. The possible mechanism of cellular uptake of supramolecular liposomes and intracellular drug release is shown in Fig. 5e. In contrast, DOX fluorescence is located at the perinuclear region instead of the nucleus when MCF-7 cells are incubated with DOX-loaded conventional liposomes for 30 min and 1 h. As it is well known that free DOX easily enters the MCF-7 cancer cells, accumulates and homogeneously distributes in the nuclei, thus, the fast accumulation of DOX in nuclei of MCF-7 cells with the incubation of DOX-loaded supramolecular liposomes suggests that DOX is quickly released due to the destruction of supramolecular liposomes in acidic cellular compartments. Based on these results, we conclude that supramolecular liposomes exhibit fast responsive ability compared to conventional covalent-bonded liposomes.

### *In vitro* cytotoxicity of supramolecular liposomes

**Fig. 6:**
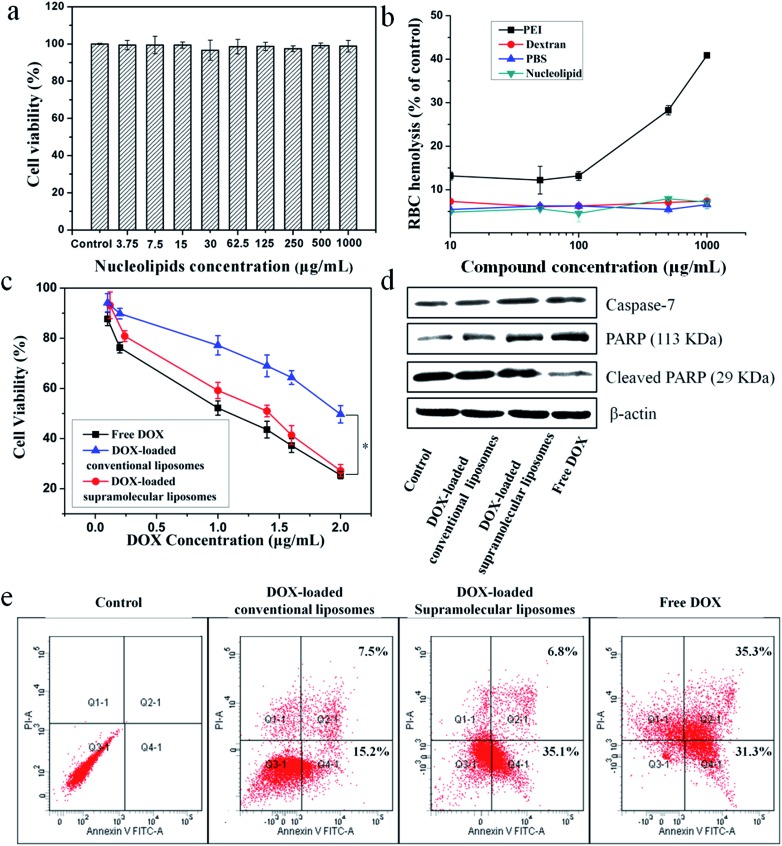
*In vitro* cytotoxicity and proliferation inhibition of supramolecular liposomes from DOA : UPC. (a) Cell viability of NIH/3T3 cells against supramolecular DOA : UPC phospholipids after being cultured for 48 h with different concentrations. (b) Hemolysis assay of supramolecular DOA : UPC phospholipids compared with PEI, PBS and dextran with different concentrations (Triton X-100 was used as a 100% hemolysis value). Data are presented as the average ± standard deviation (*n* = 3). (c) Cell viability of MCF-7 against (i) free DOX, (ii) DOX-loaded supramolecular liposomes and (iii) DOX-loaded conventional liposomes after incubation for 48 h with different DOX concentration. (d) The expression levels of caspase-7 and PARP in MCF-7 cells induced by free DOX, DOX-loaded conventional liposomes and DOX-loaded supramolecular liposomes at the same concentration (5 μg mL^–1^) for 24 h, analyzed by Western blotting. Untreated cells are set as a control, and β-actin is the loading control. (e) Flow cytometry analysis for apoptosis of MCF-7 cells induced by free DOX, DOX-loaded conventional liposomes and DOX-loaded supramolecular liposomes at the same DOX concentration of 5 μg mL^–1^ for 24 h. Lower left: living cells; lower right: early apoptotic cells; upper right: late apoptotic cells; upper left: necrotic cells. Inserted numbers in the profiles indicate the percentage of the cells present in this area.

The *in vitro* cytotoxicity of supramolecular liposomes with molecular recognition of nucleobases was evaluated by a standard MTT (3-(4,5-dimethylthiazol-2-yl)-2,5-diphenyltetrazolium bromide) assay against NIH/3T3 cells (a mouse embryonic fibroblast cell line). As shown in Fig. 6a, the cell viability after 48 h of incubation with supramolecular liposomes up to 1 mg mL^–1^ remains nearly 100% compared with the untreated cells, indicating that these supramolecular liposomes show low cytotoxicity to normal cells. The *in vitro* hemolysis assay of supramolecular liposomes was also estimated under physiological conditions *via* testing the amount of the hemoglobin released from red blood cells (RBC) in the supernatant. Dextran and polyethylenimine (PEI) were used as reference controls, and Triton X-100 (1% v/v) was used as a 100% hemolysis value. After 1 h incubation, supramolecular liposomes show comparable hemoglobin release to dextran, which is significantly lower than the value of PEI even at the concentration of 1 mg mL^–1^ (Fig. 6b), whereas PEI causes approximate 8 times higher RBC hemolysis than the supramolecular liposomes. The study exhibits negligible hemolytic activity of supramolecular liposomes. Based on the above results, supramolecular liposomes exhibit good biocompatibility.

### Higher anticancer cytotoxicity of DOX-loaded supramolecular liposomes *vs.* conventional DOX-loaded liposomes *in vitro*

The ability of drug-loaded carriers to inhibit the proliferation of tumor cells is an important consideration for cancer therapy. Thus, the *in vitro* cytotoxicity of DOX-loaded supramolecular liposomes was evaluated and compared with free DOX using MTT assays in MCF-7 cells. The conventional liposomes loaded with DOX were also evaluated under identical conditions as the control. As depicted in Fig. 6c, DOX-loaded supramolecular liposomes show slightly lower cytotoxicity to MCF-7 cancer cells when compared with free DOX; while DOX-loaded supramolecular liposomes exhibit much higher cytotoxicity than DOX-loaded conventional liposomes in all the doses tested in MCF-7 cells. In agreement with the above results, we can deduce that supramolecular liposomes with molecular recognition of nucleobases can release DOX in the cells rapidly in response to the endo/lysosomal pH and thus exhibit enhanced inhibition of the proliferation of human cancer cells.

It is well known that the anticancer drug DOX-induced tumor cell death is mainly apoptotic (programmed cell death).^52^ Here, the FITC-Annexin V/propidium iodide (PI) method was used to determine whether the death of cancer cells incubating with DOX-loaded supramolecular liposomes was induced by apoptosis. MCF-7 cells were treated with DOX-loaded supramolecular liposomes, DOX-loaded conventional liposomes and free DOX at equivalent dose of DOX (5 μg mL^–1^) for 24 h and then subjected to FITC-Annexin V/PI staining. The untreated MCF-7 cells were used as control. As shown in Fig. 6e, treatment of the cells with DOX-loaded supramolecular liposomes results in 41.9% of cells in the apoptosis phase; more precisely, ∼35.1% of cells were in the early apoptosis phase and ∼6.8% cells in the late apoptosis phase, whereas treatment with DOX-loaded conventional liposomes results in only 22.7% of cells in the apoptosis phase. In addition, the free DOX induces more apoptotic cells than the DOX-loaded supramolecular liposomes, which is in accordance with MTT analysis. In comparison with conventional liposomes, the supramolecular liposomes promote a much higher apoptotic rate of MCF-7 cells with the same dose. The enhanced apoptosis induced by DOX-loaded supramolecular liposomes is likely due to the rapid release of DOX from supramolecular liposomes under mildly acidic intracellular environment.

To further confirm this result, we analyzed poly(ADP-ribose) polymerase (PARP) and caspase-7 activation, a key effector of cell apoptosis.^53^ MCF-7 cells were treated with the above three drug formulations at equivalent dose of DOX (5 μg mL^–1^) for 24 h, and the expression levels of caspase-7 and cleaved PARP were detected by Western blot analysis. The expression level of caspase-7 is significantly up-regulated and PARP is cleaved in response to the formulations compared to the control (Fig. 6d), suggesting that the antiproliferative and cytotoxic effects of DOX could be attributed to the activity of these apoptosis mediators. Moreover, cells treated with DOX-loaded supramolecular liposomes exhibit higher expression levels of caspase-7 and PARP compared to the cells treated with the DOX-loaded conventional liposomes, indicating a better apoptosis-inducing effect of DOX-loaded supramolecular liposomes over the conventional liposomes. However, caspase-7 activity and PARP cleavage were still more apparent with free DOX, which may be explained by the relatively long-lasting release of the parent drug from supramolecular liposomes, as shown in Fig. 5c.

### *In vivo* stability and accumulation of DOX-loaded supramolecular liposomes in tumors

**Fig. 7:**
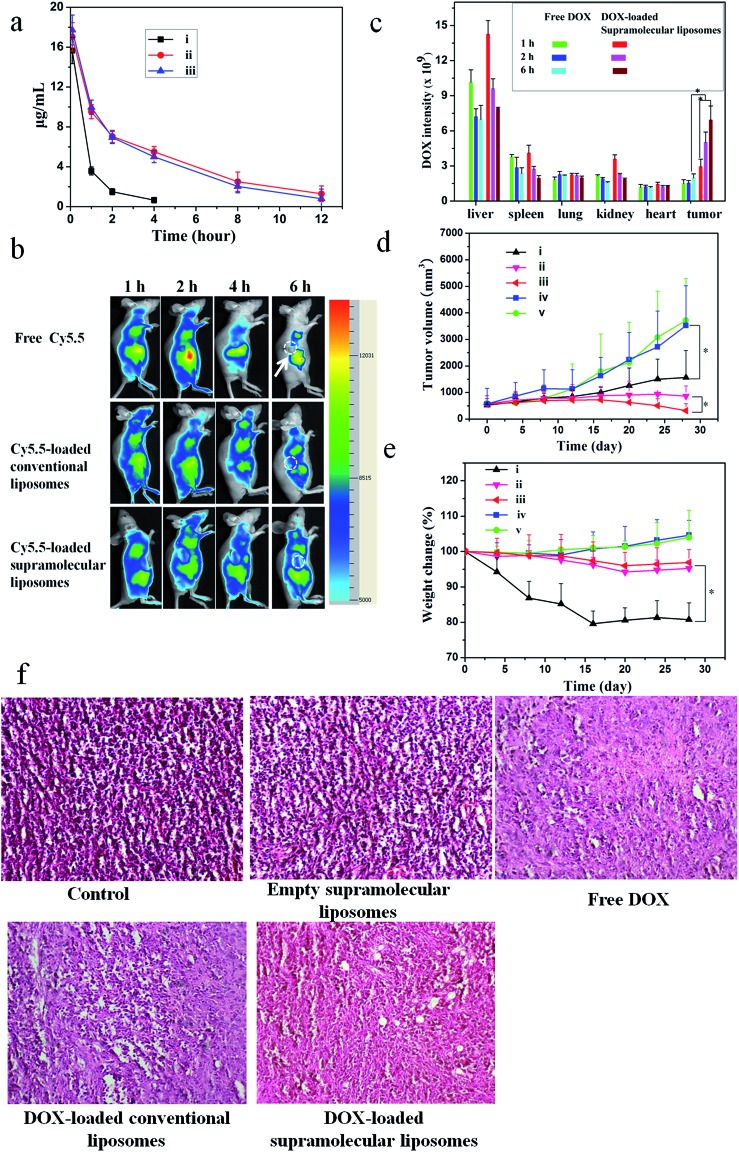
Antitumor efficacy of DOX-loaded supramolecular liposomes from nucleoside phospholipids DOA : UPC in MCF-7 tumor-bearing Balb-c/nude mice. (a) Representative plasma concentration–time profiles of (i) free DOX, (ii) DOX-loaded conventional liposomes (DOPC) and (iii) DOX-loaded supramolecular liposomes after intravenous administration in rats; DOX equivalent doses are 10 mg kg^–1^. Data are presented as the average ± standard deviation (*n* = 4). (b) *In vivo* non-invasive NIR images of free Cy5.5, Cy5.5-loaded conventional liposomes and Cy5.5-loaded supramolecular liposomes. Solid arrows indicate the tumors. (c) Biodistribution of free DOX and DOX-loaded supramolecular liposomes administrated intravenous injection to mice. Data are presented as average ± standard error (*n* = 4), and the statistical significance level is *P < 0.05. (d) Tumor volume changes with increasing time and (e) body weight changes in Balb-c/nude mice after intravenous administration of (i) free DOX, (ii) DOX-loaded conventional liposomes, (iii) DOX-loaded supramolecular liposomes, (iv) supramolecular liposomes without drug load and (v) blank control (*n* = 6) *P < 0.05. At day 20 after inoculating MCF-7 cells, each sample was injected once every 4 days for 28 days at the same doses of DOX (10 mg kg^–1^). (f) Pathological section of H&E staining of mammary cancer MCF-7 implant tumor.

A pharmacokinetic study was undertaken by intravenous injection of the free DOX, DOX-loaded conventional liposomes and DOX-loaded supramolecular liposomes to Sprague-Dawley (SD) rats (∼200 g). The time course profiles in plasma of DOX are depicted in Fig. 7a. It can be found that DOX-loaded conventional liposomes and DOX-loaded supramolecular liposomes are retained in the bloodstream for more than 12 h, suggesting the high stability of supramolecular liposomes in plasma. On the other hand, free DOX is quickly removed from the blood circulation system after intravenous injection. The longer circulation time of supramolecular liposomes facilitates the accumulation of anticancer drugs at the tumor tissue through the enhanced permeability and retention (EPR) effect. This finding is in agreement with the results of *in vivo* imaging in breast cancer xenografts in mice. *In vivo* retention assay in breast tumors was performed using free Cy5.5, Cy5.5-loaded conventional liposomes and supramolecular liposomes (Fig. 7b). Fluorescence images of Cy5.5 clearly show the fast quenching when a single dose of free Cy5.5 is given, while the fluorescence does not significantly decrease when the conventional liposomes and supramolecular liposomes with equivalent Cy5.5 dose are given. These results indicate that free drug is metabolized quickly, while the drug-loaded supramolecular liposomes have the ability of sustained drug release and thus long-term action on the tumor cells. The pharmacokinetic study and *in vivo* imaging results demonstrate that the drug-loaded supramolecular liposomes prolong the blood circulation of the drugs and accumulate at a higher accumulation in the tumors.

To determine tissue tropism of supramolecular liposomes, *in vivo* biodistribution of DOX-loaded supramolecular liposomes was evaluated in MCF-7 tumor-bearing mice after intravenous injection with different time intervals. The tumor-bearing mice treated with free DOX were used as a control. The biodistribution profiles show that DOX-loaded supramolecular liposomes are predominately localized in liver, spleen, kidney and tumor in the first 2 h. After 6 h post-injection, the content of DOX-loaded supramolecular liposomes obviously decreases in liver, kidney and spleen, whereas the upward trend in tumor is apparent (Fig. 7c). After a tail-vein injection of DOX-loaded conventional liposomes, the distribution trend of the conventional liposomes is similar to that of supramolecular liposomes (ESI Fig. S21†). Compared to that of DOX-loaded supramolecular liposomes, the concentration of free DOX is remarkably lower in the tumor. Free DOX mainly accumulates in the liver, followed by spleen, kidney, lung and heart. These data suggest that DOX-loaded supramolecular liposomes can be accumulated in tumors by passive targeting through the EPR effect.

### *In vivo* anticancer activities

To evaluate whether efficient accumulation and improved biodistribution lead to the enhancement of therapeutic efficacy, MCF-7 tumor-bearing mice were intravenously injected with free DOX, DOX-loaded supramolecular liposomes, DOX-loaded conventional liposomes, empty supramolecular liposomes, and PBS as control *via* the tail vein. Fig. 7d shows the changes in tumor volumes in the mice after intravenous administration. DOX-free groups (blank control and empty supramolecular liposomes) do not show any noticeable inhibition of tumor growth. Administration of free DOX is effective in tumor regression to some extent, but the free DOX does not demonstrate comparable efficacy to the DOX-loaded conventional liposomes or to the DOX-loaded supramolecular liposomes. Specially, DOX-loaded supramolecular liposomes are more efficacious at tumor reduction compared with DOX-loaded conventional liposomes.

Body weight loss is one of important indicators to evaluate DOX-induced toxicity. As shown in Fig. 7e, the groups of blank control and empty supramolecular liposomes show that the mice gain weight because of the rapid increase of the tumor volumes. Mice treated with free DOX at a concentration of 10 mg kg^–1^ exhibit about 20% decrease of body weight within 28 days, and appear to be weak after treatment. However, treatment with DOX-loaded supramolecular liposomes and DOX-loaded conventional liposomes results in a minimal weight loss (approximately 5%), suggesting that these drug carriers can remarkably reduce DOX toxicity to normal tissues. The high antitumor efficacy suggests that DOX-loaded liposomes could target tumor tissues *via* EPR effect due to their optimal nanoparticle size and the efficient release of DOX at the tumor acidic environments. These encouraging data merit further detailed *in vivo* studies.

To further confirm the therapeutic effect of DOX-loaded supramolecular liposomes, we performed hematoxylin–eosin (H & E) staining of pathological sections of MCF-7 tumors for all experimental groups (Fig. 7f). The negative control group (no drug treatment) shows histologic characteristics of malignant tumors, including hyperchromatic nuclei, scant cytoplasm, more nuclear pleomorphism, and more mitoses (control and empty supramolecular liposomes). Among the positive group (free DOX) and the experimental groups (DOX-loaded conventional liposomes and DOX-loaded supramolecular liposomes) in which free DOX, DOX-loaded conventional liposomes and DOX-loaded supramolecular liposomes are given respectively, the tumor cellularity is decreased in comparison to the negative control. The apoptotic phenomenon including shrinkage of tumor cells and cell separation from surrounding cells (named apoptotic body) can be observed. Large homogeneous red staining of necrotic tissue is also found in both experimental and positive groups. These results show that the DOX-loaded supramolecular liposome drug delivery system possess efficient antitumor ability.

In this study, we demonstrate that chemotherapeutic drugs DOX can be encapsulated into the supramolecular liposomes and then released in response to the mildly acidic condition of tumor sites while their biological effects *in vivo* are not altered. This is a crucial aspect for improving the delivery of chemotherapeutic drugs. Chemotherapy drugs suffer from numerous problems including poor bioavailability, rapid *in vivo* metabolism and/or excretion and nonspecific uptake by healthy cells and tissues.^54^ Often a large percentage of a cytotoxic drug administered to a patient does not reach the tumor, but is distributed throughout the body, causing the serious toxic effects associated with chemotherapy, thus reducing its therapeutic usefulness. Conventional liposomes on the nanoscale have shown excellent pharmacokinetic profiles for the delivery of chemotherapeutic agents such as DOX and are able to target the tumor site through the EPR effect,^14^–^17^ which is also confirmed in this study by the conventional liposome control. However, one major drawback of conventional liposome-based drug carriers is the lack of tunable triggers for drug release.^10^,^18^ In contrast, our supramolecular liposomes derived from supramolecularly engineered phospholipids that are composed of biocompatible materials combine the advantages of conventional nanoscale liposomes and dynamic supramolecular properties together. Our bioevaluation data including the cellular and animal levels indicate that the DOX-loaded supramolecular liposomes can efficiently accumulate in the tumor tissues and subsequently internalize into tumor cells, similar to conventional covalent-bonded liposomes. However, due to the existence of pH-sensitive multiple hydrogen bond connection of supramolecular nucleolipids, efficient release of DOX at the acidic intracellular environments has been realized, enhancing tumor-directed therapeutic efficacy greatly. Therefore, the DOX-loaded supramolecular liposomes exhibit a better *in vitro* and *in vivo* anticancer efficiency over conventional liposomes.

A simple and straightforward preparation process is required for practical large-scale generation of nanoparticles that can be loaded with multiple drugs.^55^ Our supramolecular approach is another major advantage over the multiple-step and cumbersome techniques required for covalent synthesis of conventional covalent-based phospholipids. Importantly, the linkage of complementary hydrogen bonding endows the phospholipids with strong stimuli-responsive functions, which dramatically improves the sensitivity of the resulting liposomes. The supramolecular nucleoside phospholipid library could be expanded to various new lipids by varying the combinations of chain length and species, type of nucleoside linkages, and head groups. Supramolecular nucleoside phospholipids provide a rich supplement to the existing lipid world. They may contribute new properties to expand the possible applications of lipids in biology and commerce.

The size of a nanoparticle is a critical factor that influences the cellular internalization process, as well as *in vivo* performance.^55^ An additional advantage of the supramolecular liposomes we developed is that the size can be easily manipulated by changing the hydrophobic tails of supramolecularly engineered phospholipids. This allows the carrier size to be tailored for specific therapeutic treatments.

## Conclusions

In summary, we put forward and construct an upgraded generation of phospholipids *via* the molecular recognition of complementary nucleobase pairs. Different kinds of supramolecularly engineered phospholipids are easily obtained through non-covalent coupling of the uridine-functionalized heads and the adenosine-functionalized tails. Compared to the conventional phospholipid system, the supramolecular phospholipids have the advantage of good responsive ability and facile preparation. The amphiphilic supramolecular phospholipids self-assemble into liposome-like bilayer structures with high pH-sensitive ability. Our demonstration of successfully inhibiting tumor growth in a murine cancer model using the supramolecular liposomes suggests that they have great potential for use as delivery vehicles for treatment of various types of diseases. As an extension of conventional covalent-bonded phospholipids, the supramolecularly engineered phospholipids with stimuli-responsive ability and ease of preparation may open up new perspectives in academic research and clinic applications.

## Supplementary Material

Supplementary information
