# Correction: Supramolecularly engineered phospholipids constructed by nucleobase molecular recognition: upgraded generation of phospholipids for drug delivery

**DOI:** 10.1039/c5sc90038g

**Published:** 2015-07-01

**Authors:** Dali Wang, Chunlai Tu, Yue Su, Chuan Zhang, Udo Greiser, Xinyuan Zhu, Deyue Yan, Wenxin Wang

**Affiliations:** a School of Chemistry and Chemical Engineering , State Key Laboratory of Metal Matrix Composites , Shanghai Jiao Tong University , 800 Dongchuan Road , Shanghai 200240 , People's Republic of China . Email: xyzhu@sjtu.edu.cn ; Fax: +86-21-54741297 ; Tel: +86-21-34203400; b Charles Institute of Dermatology , School of Medicine and Medical Science , University College Dublin , Belfield , Dublin 4 , Ireland . Email: wenxin.wang@ucd.ie

## Abstract

Correction for ‘Supramolecularly engineered phospholipids constructed by nucleobase molecular recognition: upgraded generation of phospholipids for drug delivery’ by Dali Wang *et al.*, *Chem. Sci.*, 2015, **6**, 3775–3787.



## 

DMA and DOA were displayed incorrectly in the graphical abstract and Fig. 1 and 3. The corrected figures are shown below.

**Fig. 1:**
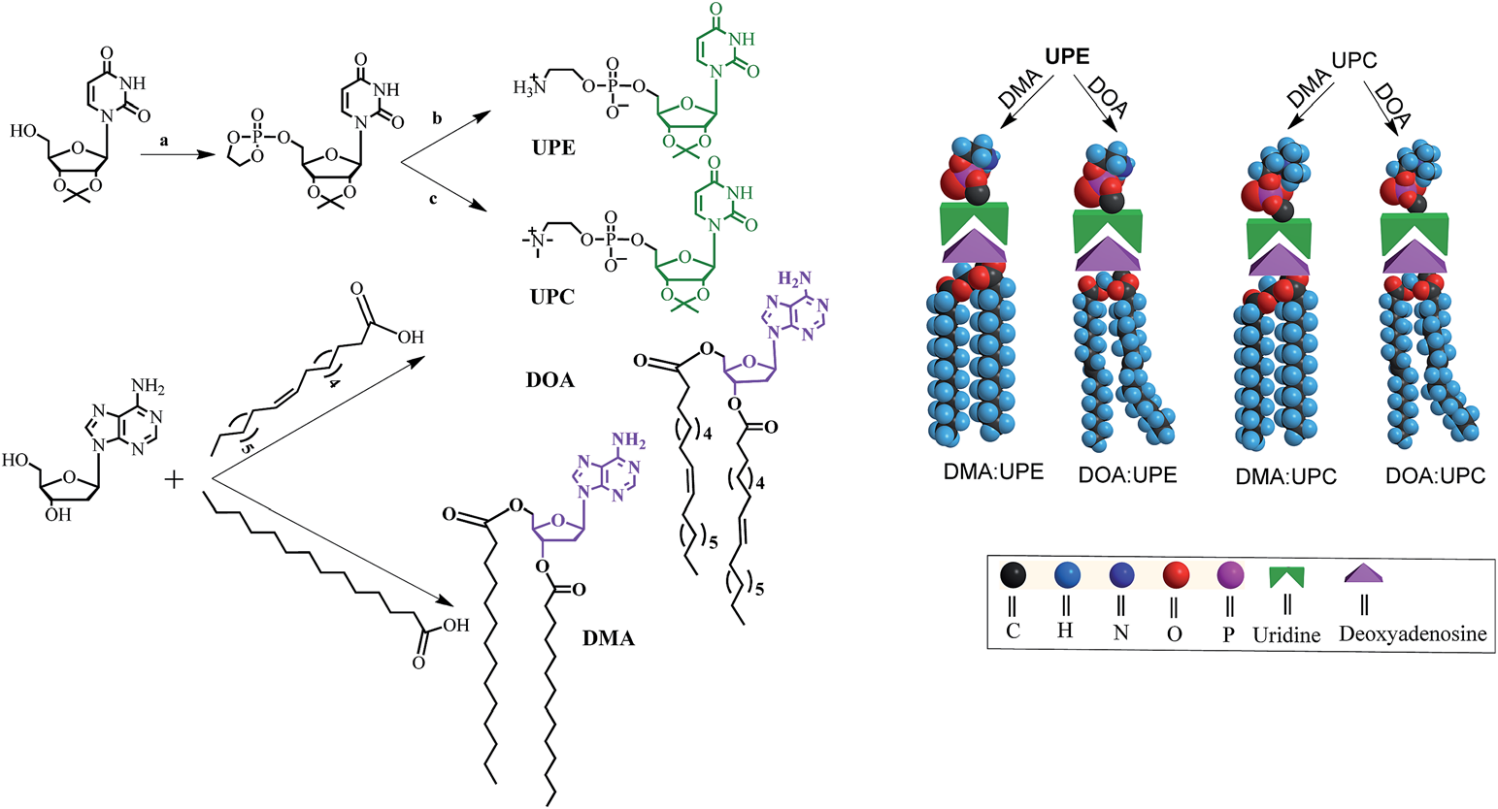
Synthetic route, chemical structures of nucleoside phospholipids and schematic representation for the formation of supramolecular phospholipids. Reagents and conditions: (a) chlorooxodioxaphospholane, TEA, THF, 0 °C, 15 h; (b) trimethylamine, acetonitrile, THF, 60 °C, 24 h. (c) Ammonia, acetonitrile, THF, 65 °C, 48 h. UPE and UPC are uridine-functionalized PE and PC as hydrophilic phospholipid head, respectively. DMA and DOA are adenosine-functionalized myristic acid and oleic acid as hydrophobic tails, respectively. Through the molecular recognition between adenosine and uridine, these two components form four different types of supramolecular nucleoside phospholipids (DMA : UPE, DOA : UPE, DMA : UPC and DOA : UPC) by mixing a uridine-terminated head and an adenosine-terminated tail.

**Fig. 3:**
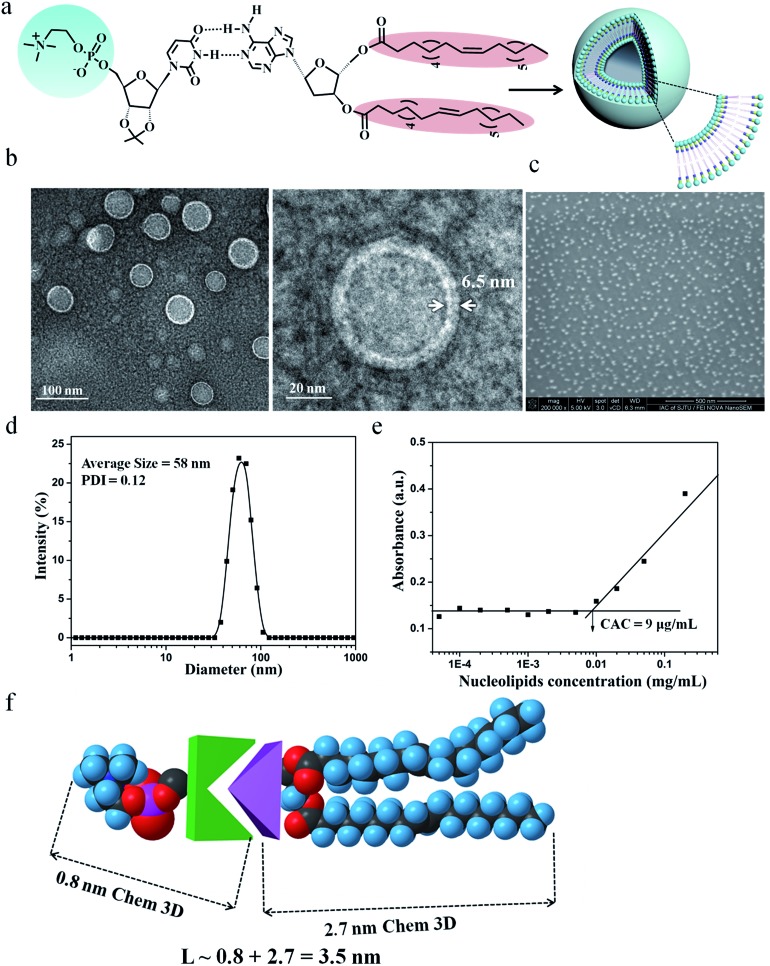
Characterization of molecular self-assembly of supramolecular nucleoside phospholipids DOA : UPC. (a) Schematic representation of a supramolecular liposome self-assembled from the DOA : UPC nucleoside phospholipids. Supramolecular nucleoside phospholipids self-assemble into liposome-like bilayer structures in aqueous solution. (b) Representative TEM images of negatively stained supramolecular DOA : UPC liposomes. The liposome wall thickness is about 6.5 nm. (c) Representative SEM image of supramolecular DOA : UPC liposomes (scale bars are 500 nm). (d) DLS profile for the supramolecular liposomes. (e) Relationship of the absorbance and the concentration of DOA : UPC in aqueous solutions (*λ* = 313 nm, 25 °C). (f) Estimation of the length of an extended DOA : UPC molecule according to the Chem3D results.

Graphical abstract:
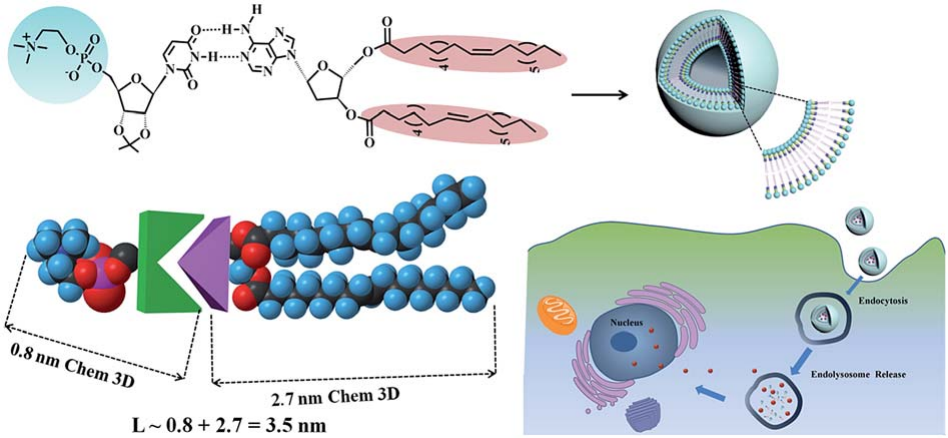


The Royal Society of Chemistry apologises for these errors and any consequent inconvenience to authors and readers.

