# Supplementary material for: Supramolecularly engineered phospholipids constructed by nucleobase molecular recognition: upgraded generation of phospholipids for drug delivery
Source: Chem Sci. 2015 May 12;6(7):3775–87. doi: 10.1039/c5sc01188d (PMC5707505; doi:10.1039/c5sc01188d)
Supplement: Supplementary file 1 [file SC-006-C5SC01188D-s001.pdf]

Electronic Supplementary Material (ESI) for Chemical Science.
This journal is © The Royal Society of Chemistry 2015

Supplementary Information

Supramolecularly engineered phospholipids constructed by nucleobase molecular recognition: upgraded generation of phospholipids for drug delivery

Dali Wang,[a] Chunlai Tu,[a] Yue Su,[a] Chuan Zhang,[a] Udo Greiser,[b] Xinyuan Zhu,[*,a] Deyue Yan,[a] and Wenxin Wang[*,b]

[a] *School of Chemistry and Chemical Engineering, State Key Laboratory of Metal Matrix Composites, Shanghai Jiao Tong University, 800 Dongchuan Road, Shanghai 200240, P. R. China*

[b] *Charles Institute of Dermatology, School of Medicine and Medical Science, University College Dublin, Belfield, Dublin 4, Ireland*

*Correspondence to: xyzhu@sjtu.edu.cn; wenxin.wang@ucd.ie

Table of Content

1. **Materials** **S3**

2. **Measurements** **S3**

3. **Methods** **S5**

3.1 Synthesis of uridine acetonide phosphatidylcholine (UPC) S5

3.2 Synthesis of uridine acetonide phosphatidylethanolamine (UPE) S6

3.3 Synthesis of 3',5'-dimyristoyladenosine (DMA). S7

3.4 Synthesis of 3',5'-dioleoyladenosine (DOA) S8

3.5 Fabrication of supramolecular liposomes and conventional liposomes. S9

3.6 Preparation of DOX-loaded supramolecular liposomes and DOX-loaded conventional liposomes. S10

3.7 *In vitro* release measurements. S11

3.8 Cell culture S12

3.9 Biocompatibility of supramolecular liposomes S12

3.10 Intracellular drug release S13

3.11 *In vitro* cytotoxicity evaluation S14

3.12 Apoptosis analysis with flow cytometry S15

3.13 Western blotting analysis. S15

3.14 *In vivo* biodistribution and tumor targeting capability of DOX-loaded supramolecular liposomes in tumor-bearing mice S16

3.15 Pharmacokinetic studies S17

3.16 *In vivo* antitumor efficacy S17

3.17 Statistics S19

4. **Figures** **S19**

5. **References** **S40**

1. Materials

1,2-Dioleoyl-sn-glycero-3-phosphocholine (DOPC) was purchased from Sigma and used as received. Uridine acetonide, 2-chloro-2-oxo-1,3,2-dioxaphospholane, 2'-deoxyadenosine, oleic acid and 3-(4,5-dimethyl-thiazol-2-yl)-2,5-diphenyl tetrazolium bromide (MTT) were purchased from Sigma-Aldrich and used without further purification. Trimethylamine (ca. 13% in tetrahydrofuran (THF), ca. 2 mol/L) and myristic acid were used as purchased from TCI. 1-Ethyl-3-(3-dimethyllaminopropyl)carbodiimide hydrochloride (EDC) and 4-dimethylamino-pyridine (DMAP) were purchased from Aladdin. THF was dried by refluxing with the fresh sodium-benzophenone complex under N_2 and distilled just before use. Triethylamine (TEA) and dichloromethane were treated with calcium hydride and distilled before use. Doxorubicin hydrochloride (DOX) was purchased from Beijing Huafeng United Technology Corporation and used as received. Clear polystyrene tissue culture treated 12-well and 96-well plates were obtained from Corning Costar. β-Actin polyclonal antibodies and Caspase-7 polyclonal antibodies were purchased from Cell Signaling Technology (Danvers, MA, USA). PARP polyclonal antibodies were purchased from Abcam (Hong Kong) Ltd. All other reagents and solvents were purchased from the domestic suppliers and used as received unless mentioned.

2. Measurements

Nuclear magnetic resonance (NMR). All NMR spectra were recorded on Bruker AVANCEIII 400 spectrometer with deuterium oxide (D_2O), dimethylsulfoxide-d_6 (DMSO-d_6) or deuterated chloroform ($CDCl_3$) as solvents (^1H at 400 MHz, ^{13}C at 100

MHz and ^{31}P at 162 MHz).

Mass spectrometry (MS). MS experiments were carried out on a Waters Premier Q-TOF, employing electrospray ionization in positive mode.

Dynamic light scattering (DLS). DLS measurements were performed with a Malvern Zetasizer Nano S apparatus equipped with a 4.0 mW laser operating at ë = 633 nm. All samples were measured at a scattering angle of 173°. The data were the mean of three tests.

Transmission electron microscopy (TEM). The structure and size of liposomes were characterized by a JEOL JEM-100CX-II instrument at a voltage of 200 kV. To observe the vesicles' structure, 500 ìL of solution containing liposomes was spread on parafilm and mixed with 500 ìL of 1 wt% sodium tungstate (maintained at pH 6.8 with 1.0 N KOH) (liposome dispersion/sodium tungstate in 1:1 v/v ratio), a TEM negative stain. After keeping for 2 min, a drop of this solution was placed on carbon-coated copper grid and stabilized with carbon film coating. The excess fluid was drained off with filter paper. These TEM grids were then floated on top of deionized water to remove any excess stain. The grids were then wicked and allowed to dry at room temperature before measurement.

Scanning electron microscopy (SEM). SEM measurements were performed on a SU8020 (Hitachi) and NOVA NanoSEM 230 (FEI). The samples for SEM observation were prepared by depositing several drops of the solution (1 mg/mL) onto the surface of clean glass, and the samples were air-dried at room temperature for 24 h. The samples were coated with a thin film of gold before measuring.

3. Methods

3.1 Synthesis of uridine acetonide phosphatidylcholine (UPC)

Uridine acetonide (0.80 g, 2.81 mmol) was dissolved in freshly distilled THF (30 mL) and dry TEA (803 µL, 2 eq, 5.71 mmol) was added under nitrogen. The reaction mixture was placed on an ice bath. 2-Chloro-2-oxo-1,3,2-dioxaphospholane (414 µL, 1.6 eq, 4.5 mmol) was slowly added to the mixture while keeping the mixture on an ice bath. Then the reaction mixture was stirred at room temperature for 15 h. Next, the TEA salts were removed by filtration under vacuum at 0 °C. Most of the solvent was evaporated under reduced pressure at 0 °C and 10 mL of the residual solution was used directly without further purification in the following step.

Trimethylamine (2 M solution in THF, 22 mL, 44 mmol) was added to a pressure tube at -78 °C. Then, 10 mL of cold (-20 °C) dry acetonitrile and the solution containing the uridine-oxo-dioxaphospholane were added to the cold trimethylamine solution. The reaction mixture was stirred at 60 °C for 48 h. The product was precipitated in the sealed tube. After evaporation of the solvent at room temperature, the supernatant was removed, and the precipitate obtained was washed in dry acetonitrile. 1.0 g of a hygroscopic solid was obtained after drying under high vacuum (Yield: 80 %). ^1H NMR (400 MHz, DMSO-d_6, 298 K) δ ppm: 1.28 (s, 3H, CH_3), 1.48 (s, 3H, CH_3), 3.11 (s, 9H, $N(CH_3)_3$), 3.50 (t, 2H, NCH_2), 3.79 (t, 2H, CH_2O), 4.02 (m, 2H, $H_{5'}$), 4.15 (m, 1H, $H_{4'}$), 4.79 (m, 1H, $H_{2'}$ or $H_{3'}$), 4.91 (m, 1H, $H_{2'}$ or $H_{3'}$), 5.59 (d, 1H, H_5), 5.84 (d, 1H, $H_{1'}$), 7.84(d, 1H, H_6), 11.40 (s, 1H, NH). ^{13}C NMR (100 MHz, DMSO-d_6, 298 K) δ: 25.84 (CH_3), 27.70 (CH_3), 53.79 ($N(CH_3)_3$), 59.12 (CH_2O), 65.17 (C5’), 66.10 (N^+CH_2), 81.51 ($C_{3'}$), 84.16 ($C_{2'}$), 85.89 ($C_{4'}$), 92.04 ($C_{1'}$), 102.57

(C_5), 113.69 (OCO), 142.94 (C_6), 151.04 (C_2), 163.88 (C_4). ^{31}P NMR (162 MHz, D_2O): δ: 0.10 ppm. HRMS (ESI/Q-TOF) MH^+ (theoretical = 450.1655, observed = 450.1627).

3.2 Synthesis of uridine acetonide phosphatidylethanolamine (UPE)

UPE was synthesized using two different procedures.

Procedure 1. Synthesis of 2 was carried out following the same procedure as described above. Then 20 mL of dry acetonitrile and a solution of uridine-oxo-dioxaphospholane in 10 mL of anhydrous THF were placed in a pressure tube cooled at -78 °C. Subsequently, 87 mL of a solution of ammonia in THF (0.5 M, 43.5 mmol) was added. The pressure tube was sealed and then heated in an oil bath at 65 °C for 48 h. The product was precipitated in the sealed tube. After evaporation of the solvent at room temperature, the supernatant was removed, and the precipitate obtained was washed in dry acetonitrile. 0.9 g of a hygroscopic solid was obtained after drying under high vacuum (Yield: 78 %).

Procedure 2. Phosphorus oxychloride (0.80 g, 2.81 mmol) was dissolved in freshly distilled THF (10 mL) and dry TEA (803 μL, 2 eq, 5.71 mmol) was added under nitrogen. The reaction mixture was placed on an ice bath. A solution of uridine acetonide (0.80 g, 2.81 mmol) in freshly distilled THF (30 mL) was added slowly to the mixture while keeping the mixture on an ice bath. Then the reaction mixture was stirred at room temperature for 2 h. Next, the TEA salts were removed by filtration. Most of the solvent was evaporated under reduced pressure at 0 °C and 8 mL of the residual solution was used directly without further purification in the following step.

A solution of ethanolamine (0.19 g, 3.1 mmol) and TEA (1.6 mL, 11.2 mmol) in 10 mL freshly distilled THF was added dropwise to a solution of phosphatidic acid dichloride (5) in 8 mL THF at 10 °C. Then the reaction mixture was stirred at 20 °C for 0.5 h. Next, the reaction mixture was filtered to remove precipitated TEA salts. Most of the solvent was removed under reduced pressure and the residue was precipitated into hexane and dried under vacuum to a constant weight.

A solution of 6 in 20 mL of 2-propanol was mixed with 10 mL of acetic acid (20% solution in water). After 2 h, most of the solvent was removed under reduced pressure. The product (4) was purified by chromatography (reverse phase, methanol/H_2O: 80/20). 0.4 g of a hygroscopic solid was obtained after drying under high vacuum (Yield: 34 %).

Characterisation data for UPE. ^{1}H NMR (400 MHz, DMSO-d_6, 298 K) δ ppm: 1.27 (s, 3H, CH_3), 1.47 (s, 3H, CH_3), 3.44 (t, 2H, NH_3CH_2), 3.58 (t, 2H, CH_2O), 3.87 (m, 2H, $H_{5'}$), 4.16 (m, 1H, $H_{4'}$), 4.79 (m, 1H, $H_{2'}$ or $H_{3'}$), 4.89 (m, 1H, $H_{2'}$ or $H_{3'}$), 5.58 (d, 1H, H_5), 5.84 (d, 1H, $H_{1'}$), 7.25 (s, 3H, NH_3^+), 7.84 (d, 1H, H_6), 11.32 (s, 1H, NH). ^{13}C NMR (100 MHz, DMSO-d_6, 298 K) δ: 25.86 (CH_3), 27.72 (CH_3), 62.35 ($NH_3^+CH_2$), 65.05 (CH_2O), 66.88 (C5’), 81.47 ($C_{3'}$), 84.17 ($C_{2'}$), 85.75 ($C_{4'}$), 91.80 ($C_{1'}$), 102.60 (C_5), 113.67 (OCO), 142.72 (C_6), 151.05 (C_2), 163.85 (C_4). ^{31}P NMR (162 MHz, D_2O): δ: 1.06 ppm. HRMS (ESI/Q-TOF) MH^+ (theoretical = 408.1172, observed = 408.1183).

3.3 Synthesis of 3’,5’-dimyristoyladenosine (DMA)

2’-Deoxyadenosine (0.50 g, 1.86 mmol) was suspended in anhydrous

dichloromethane (25 mL). Then, EDC (0.86 g, 4.46 mmol) was added, followed by myristic acid (1.02 g, 4.46 mmol) and DMAP (0.27 g, 2.23 mmol). The mixture was stirred at room temperature for 24 h. The mixture was then filtered and extracted twice with water, dried with Na_2SO_4. The solvent was evaporated on a rotary evaporator. The rude product was purified by flash chromatography on a silica column by using a dichloromethane:methanol (20:1) eluent system (Yield: 67 %). ^{1}H NMR (400 MHz, $CDCl_3$, 298 K) δ ppm: 8.35 (s, 1H), 7.99 (s, 1H), 6.45-6.40 (dd, 1H), 5.85 (s, 2H), 5.36-5.31 (m, 1H), 4.39-4.32 (m, 3H), 2.93-2.88 (m, 1H), 2.65-2.60 (m, 1H), 2.38-3.31 (m, 4H), 2.20-1.94 (m, 4H), 1.65-1.58 (m, 4H), 1.30-1.26 (m, 40H), 0.90-0.84 (t, 6H). ^{13}C NMR (100 MHz, DMSO-d_6, 298 K) δ: 173.26 & 173.10 (C11 & C25), 156.77 (C1), 153.26 (C4), 149.80 (C3), 138.72 (C5), 119.91 (C2), 84.20 (C6), 82.23 (C9), 74.89 (C8), 63.99 (C10), 35.96 (C7), 34.09 & 33.96 (C12, C26), 31.99 (C22, C36), 29.73 & 29.09 (C14-C21, C28-C35), 25.00 (C13, C27), 22.78 (C23, C37), 14.60 (C24, C38) with carbon atoms as labeled in Figure S4. HRMS (ESI/Q-TOF): MH^+ (theoretical = 672.5064, observed = 672.5043).

3.4 Synthesis of 3',5'-dioleoyladenosine (DOA)

2'-Deoxyadenosine (0.50 g, 1.86 mmol) was suspended in anhydrous dichloromethane (25 mL). Then, EDC (0.86 g, 4.46 mmol) was added, followed by oleic acid (1.26 g, 4.46 mmol) and DMAP (0.27 g, 2.23 mmol). The mixture was stirred at room temperature for 20 h. The mixture was then filtered and extracted twice with water, dried with Na_2SO_4. The solvent was evaporated on a rotary evaporator, yielding 1.48 g of crude product. The dried product was dissolved in

dichloromethane (6 mL). This solution (3 mL) was purified by flash chromatography on a silica column by using a dichloromethane:methanol (20:1) eluent system (Yield: 60%). ^{1}H NMR (400 MHz, $CDCl_3$, 298 K) δ ppm: 8.33 (s, 1H), 8.0 (s, 1H), 6.45-6.40 (dd, 1H), 5.98 (s, 2H), 5.36-5.31 (m, 5H), 4.39-4.32 (m, 3H), 2.65-2.60 (m, 1H), 2.38-3.31 (m, 5H), 2.20-1.94 (m, 8H), 1.65-1.58 (m, 4H), 1.30-1.26 (m, 40H), 0.90-0.84 (t, 6H). ^{13}C NMR (100 MHz, $CDCl_3$, 298 K) δ: 173.44 & 173.35 (C11 & C29), 155.66 (C1), 153.09 (C4), 149.76 (C3), 138.72 (C5), 130.27-129.89 (4s, C19, C20, C37, C38), 120.17 (C2), 84.80 (C9), 82.96 (C6), 74.53 (C8), 63.84 (C10), 38.26 (C7), 34.36 & 34.27 (C12, C30), 29.98 & 29.91 (C26, C24), 29.82-29.16 (m, C15-C18, C21-C25, C33-C36, C39-C43), 27.44 & 27.39 (C14, C32), 25.02 (C13, C31), 22.90 (C27, C45), 14.34 (C28, C46) with carbon atoms as labeled in Figure S5. HRMS (ESI/Q-TOF): MH^+ (theoretical = 780.6131, observed = 780.6115).

3.5 Fabrication of supramolecular liposomes and conventional liposomes

Four kinds of supramolecular liposomes were prepared by mixing equimolar amounts of each component (DMA/UPC, DOA/UPC, DMA/UPE and DOA/UPE) in chloroform/methanol and removing the solvent under reduced pressure. This procedure was repeated for three times, and the resultant complexes were finally dried at *in vacuo*. Take supramolecular DOA/UPC liposomes for example. Briefly, DOA (7.8 mg) and UPC (4.5 mg) were dissolved in 3 mL of chloroform/methanol (1:1, v/v) in a round-bottomed flask. The solution was evaporated and dried on vacuum for 6 h. Ultra pure water (6 mL) was added to the flask in an ultrasonic bath (Sheng Yan SCQ

3201, 40 kHz, power output 300 W). After a bath sonication for 30 min, an equal volume of PBS (40 mM, pH = 7.4) was added and sonicated for another 5 min. Similarly, conventional liposomes were prepared with DOPC in the same procedure.

The critical aggregation concentration (CAC) of supramolecular DOA/UPC nucleoside phospholipids was determined using 1,6-diphenyl-1,3,5-hexatriene (DPH) as UV probe by monitoring the absorbance at 313 nm. The concentration of supramolecular nucleolipids was varied from 5.0×10^{-5} to 0.2 mg/mL and the DPH concentration was fixed at 5.0×10^{-6} mol/L. The absorbance spectra of all solutions were recorded using Perkin-Elmer Lambda 20/2.0 UV/Vis spectrometer.

3.6 Preparation of DOX-loaded supramolecular liposomes and DOX-loaded conventional liposomes

The supramolecular DOA:UPC dry-film was prepared in a round-bottomed flask as above mentioned. A predetermined amount of DOX was dissolved in PBS buffer and then added to the flask in an ultrasonic bath (Sheng Yan SCQ 3201, 40 kHz, power output 300 W). Subsequently, the resulting solution was sonicated for 30 min, allowing the lipids to self-assemble into drug-loaded liposomes. The drug-loaded liposomes were purified according to previous report.[1] DOX-loaded liposomes were separated from free DOX by gel filtration using spin Sephadex G50 columns. Sephadex G50 was presoaked in the isoosmotic KCl buffer with 10 mM HEPES and 1 mM EDTA at pH 7.4. The other three kinds of DOX-loaded supramolecular liposomes and DOX-loaded conventional liposomes were prepared using the same method. The contents inside the dialysis tube were filtered and lyophilized. The

amount of DOX was determined with fluorescence (QM/TM/IM Steady-State & Time-Resolved Fluorescence Spectrofluorometer) measurement (excitation at 480 nm). To determine the total loading of the drug, DOX-loaded liposomes were dissolved in DMSO and analyzed with fluorescence spectroscopy, wherein calibration curve was obtained with DOX/DMSO solutions with different DOX concentrations.

Drug loading content (DLC) and drug loading efficiency (DLE) were calculated according to the following formula:

$$\text{DLC (wt\%)} = (\text{weight of loaded drug/weight of polymer}) \times 100\%$$

$$\text{DLE (\%)} = (\text{weight of loaded drug/weight of drug in feed}) \times 100\%$$

3.7 *In vitro* release measurements

A total of 6 mL of DOX-loaded liposomes was transferred to a dialysis bag with a molecular weight cutoff of 2000 Da. It was immersed in 50 mL of phosphate buffer (pH 7.4) or acetate buffer (pH 5.0) solutions with gentle shaking (100 rpm) at 37 °C in a laboratory shaker. At predetermined time intervals, 2.0 mL buffer solution outside the dialysis bag was extracted, and it was replaced by an equal volume of fresh media to keep the sink condition. The amount of released DOX was analyzed with fluorescence measurement (QM/TM/IM Steady-State & Time-Resolved Fluorescence Spectrofluorometer, excitation at 480 nm). Each experiment was done in triple and the results were the average data.

3.8 Cell culture

MCF-7 cells (a human breast adenocarcinoma cell line) and NIH/3T3 normal cells (a mouse embryonic fibroblast cell line) were cultured in DMEM (Dulbecco's

modified Eagle's medium) supplied with 10% FBS (fetal bovine serum) and antibiotics (50 units/mL penicillin and 50 units/mL streptomycin) at 37 °C in a humidified atmosphere containing 5% CO_2.

3.9 Biocompatibility of supramolecular liposomes

The relative cytotoxicity of supramolecular liposomes with molecular recognition of nucleobases was estimated by MTT viability assay against NIH/3T3 cells and hemolysis assay.

MTT assay: NIH/3T3 cells were seeded into 96-well plates at 8×10^3 cells per well in 200 μL DMEM. After 24 h incubation, the culture medium was removed and replaced with 200 μL DMEM containing serial dilutions of supramolecular liposomes. The cells were grown for another 48 h. Then, 20 μL of 5 mg/mL MTT assays stock solution in phosphate buffered saline (PBS) was added to each well. After incubating the cells for 4 h, the medium containing unreacted MTT was removed carefully. The obtained blue formazan crystals were dissolved in 200 μL per well DMSO and the absorbance was measured in a BioTek® SynergyH4 at a wavelength of 490 nm.

Hemolysis assay of supramolecular liposomes: 10 mL of blood from the ear artery of a male New Zealand white rabbit was collected. Red blood cell (RBC, 2% w/v) solution was prepared and centrifuged at 2000 rpm for 10 min at 4 °C. The plasma supernatant was removed, and the erythrocytes were resuspended in ice cold PBS. The cells were again centrifuged at 2000 rpm for 10 min at 4 °C. This was repeated more than two times to ensure the removal of any released hemoglobin. After the supernatant was removed, the cells were resuspended in PBS to get a 2% w/v RBC

solution. The nucleolipid and the reference polymers were also prepared at serial concentration (0.02, 0.1, 0.2, 1 and 2 mg/mL) with PBS (pH=7.4). Then 2 mL of the nucleolipid or the reference polymers (Dextran and PEI) prepared in PBS was added to 2 mL of the 2% w/v RBC solution in centrifuge tubes and incubated for 1 h at 37 °C. Complete hemolysis was attained using a 2% v/v Triton-X, yielding the 100% control value. After incubation, the centrifuge tubes were centrifuged, and the supernatants were transferred to the quartz cuvettes of spectrophotometer. The release of hemoglobin was determined by spectrophotometric analysis of the supernatant at 545 nm. Results were expressed as the amount of hemoglobin release induced by the conjugates as a percentage of the total.

3.10 Intracellular drug release

The experiments of intracellular drug release were performed on flow cytometry and confocal laser scanning microscopy (CLSM).

Flow Cytometry: MCF-7 cells were seeded in six-well plates at 5×10^5 cells per well in 1 mL complete DMEM and cultured for 24 h. Then the DOX-loaded supramolecular liposomes dissolved in DMEM culture medium at a final DOX concentration of 8 μg/mL were added to different wells and the cells were incubated at 37 °C for 5, 15, 30, and 60 min. Thereafter, culture medium was removed, and cells were washed with PBS twice and treated with trypsin. Subsequently, 2 mL of PBS was added to each culture well, and the solutions were centrifugated for 5 min (1000 rpm). After the supernates were removed, the cells were resuspended in 0.5 mL of PBS. Data for 1.0×10^4 gated events were collected and analysis was performed by

means of a BD FACSCalibur flow cytometer and CELLQuest software.

CLSM: MCF-7 cells were seeded in six-well plates at 2×10^5 cells per well in 1 mL complete DMEM and cultured for 24 h, followed by removing culture medium and adding DOX-loaded micelles (1 mL DMEM medium) at a final DOX concentration of 8 μg/mL. The cells were incubated at 37 °C for predetermined intervals. The cells were stained by LysoSensor Green DND-189 for 45 min at 37 °C. Then the culture medium was removed and cells were washed with PBS for three times. Subsequently, the cells were fixed with 4% paraformaldehyde for 30 min at room temperature, and the slides were rinsed with PBS for three times. Finally, the cells were stained with Hoechst 33342 for 8 min and the slides were rinsed with PBS for three times. The slides were mounted and observed by a LSM 510META.

3.11 *In vitro* cytotoxicity evaluation

The cytotoxicity of DOX-loaded supramolecular liposomes, DOX-loaded conventional liposomes and free DOX against MCF-7 cells was evaluated *in vitro* by MTT assay. MCF-7 cells were seeded into 96-well plates at an initial density of 6×10^3 cells per well in 200 μL of medium. After incubation for 24 h, the culture medium was replaced with fresh one, and the cells were treated with DOX-loaded liposome solution at a predetermined concentration. The cells were grown in a humidified environment with 5% CO_2 at 37 °C for another 48 h. After the incubation, the culture medium was removed and washed with PBS twice. Then 200 μL of DMEM and 20 μL of 5 mg/mL MTT assays stock solution in PBS were added to each well. After incubating the cells for 4 h, the medium containing unreacted dye was removed

carefully. The obtained blue formazan crystals were dissolved in 200 μL per well DMSO and the absorbance was measured in a BioTek® SynergyH4 at a wavelength of 490 nm.

3.12 Apoptosis analysis with flow cytometry

MCF-7 cells were exposed to DOX-loaded supramolecular liposomes, DOX-loaded conventional liposomes and free DOX at equivalent DOX doses (5 μg/mL) for 24 h. After that, both floating and attached cells were collected, washed three times with ice-cold PBS, and incubated at 37 °C for 15 min with Annexin V-FITC and propodium iodide (PI) to determine cell apoptosis. The samples were analyzed by flow cytometry (BD LSRFortessa, USA).

3.13 Western blotting analysis

MCF-7 cells were seeded in 6-well plates at a density of 5.0×10^5 cells per well in 2 mL of DMEM complete medium and allowed to attach for 24 h. The cells were treated with DOX-loaded supramolecular liposomes, DOX-loaded conventional liposomes and free DOX at a final DOX concentration of 5 μg/mL for 24 h. MCF-7 cells untreated were used as a negative control. After treatment for 24 h, the MCF-7 cells were harvested. Protein content in the extracts was quantified using a bicinchoninic acid (BCA) protein assay kit (Pierce, Germany). Equal amounts of proteins (30 μg/lane) were separated on SDS-PAGE and electrotransferred to 0.22 μm PVDF membranes. The membranes were then blocked with 5% non-fat dry milk in TBST (Tris buffered saline supplemented with 0.05% Tween-20) and probed with antibodies against β-actin (1:1000 dilution), caspase-7 (1:1000 dilution), PARP (1:400

dilution) followed by HRP-conjugated anti-rabbit immunoglobulin-G (IgG; 1:5000 dilution). Protein bands were detected by chemiluminescence using the ECL Western blotting substrate (Themo Scientific, USA)) according to the manufacturer's protocol and analyzed using the ChemiDoc™ MP Imaging System (Bio-Rad, USA).

3.14 *In vivo* biodistribution and tumor targeting capability of DOX-loaded supramolecular liposomes in tumor-bearing mice

All animal experiments were performed in accordance with the principles of care and use of laboratory animals, and were approved by the Animal Ethics Committee of Shanghai Jiao Tong University. Non-invasive optical imaging systems were used to observe the real-time distribution and tumor accumulation ability of fluorescent Cy5.5-loaded supramolecular liposomes prepared by film dispersion method. For *in vivo* imaging experiments, MCF-7 cells were induced in male Balb/c nude mice (4 weeks old, Chinese Academy of Sciences of Shanghai) by subcutaneous injection of 2.0×10^6 cells suspended in PBS. When the tumor volume reached approximately 250-300 mm^3, the mice were administered with free Cy5.5, Cy5.5-loaded supramolecular liposomes and Cy5.5-loaded conventional liposomes via tail vein injection, and scanned at 1, 2, 4 and 6 h using a Kodak multimode imaging system. Biodistributions of free DOX, DOX-loaded conventional liposomes and DOX-loaded supramolecular liposomes were monitored after the final intravenous injection. Mice were killed, tumors and other organs were removed and the biodistributions of these formulations were analyzed using a Kodak Image System.

3.15 Pharmacokinetic studies

SD rats (190-210 g) were chosen to examine the pharmacokinetics of DOX-loaded supramolecular liposomes, DOX-loaded conventional liposomes and free DOX. Rats were randomly divided into four groups (n = 4). DOX-loaded supramolecular liposomes, DOX-loaded conventional liposomes and free DOX solutions were intravenously administrated through the tail vein at a dose of 10 mg/kg (DOX equivalent doses), respectively. The blood samples (0.5 mL) were collected from the plexus venous in the eyeground at 15 min, 1 h, 2 h, 4 h, 8 h, and 12 h. The plasma was obtained by centrifugation at 3000 rpm for 15 min and stored at -20 °C. We treated 200 μL of plasma three times with 2 mL chloroform/ethanol mixture (4:1, v/v) in a glass test tube. After centrifugation at 3,000 rpm for 10 min, the organic layer was transferred to a test tube. The combined extract was concentrated at below 30 °C under nitrogen. The dried sample was dissolved in 2 mL of chloroform-methanol and supplemented with 1 mL of an internal standard solution (daunorubicin) and evaporated to dryness. On the basis of HPLC, the amounts of DOX were determined from standard curves previously obtained by analysis of blood samples containing known amounts of DOX.

3.16 *In vivo* antitumor efficacy

The tumors were produced in Balb/c male nude mice as described above. Mice were inspected for viewing the tumor appearance by observation and palpation. Tumor-bearing mice were randomly divided into 5 groups (each 6 mice): 1) the control group; 2) supramolecular liposomes; 3) free DOX (10 mg/kg); 4) DOX-loaded conventional liposomes (DOX, 10 mg/kg); 5) DOX-loaded supramolecular liposomes

(DOX, 10 mg/kg). Each sample was injected via the lateral tail vein once every 4 days for 28 days. The volume of tumors and weight of mice were measured before every treatment and the fourth day after the last administration to mice. Antitumor activity was evaluated in terms of tumor size ($V = 1/2ab^2$; a, long diameter; b, short diameter) by measuring two orthogonal diameters at various time points. Animals were sacrificed by cervical dislocation. Tumors were dissected and fixed with formalin for pathological section.

3.17 Statistics

All experiments were repeated at least three times. Data are presented as means ± standard deviation. Statistical significance ($p<0.05$) was evaluated by using Student's t-test when only two groups were compared. If more than two groups were compared, evaluation of significance was performed using one-way analysis of variance (ANOVA) followed by Bonferroni's post hoc test. In all tests, statistical significance was set at $p<0.05$.

4. Figures

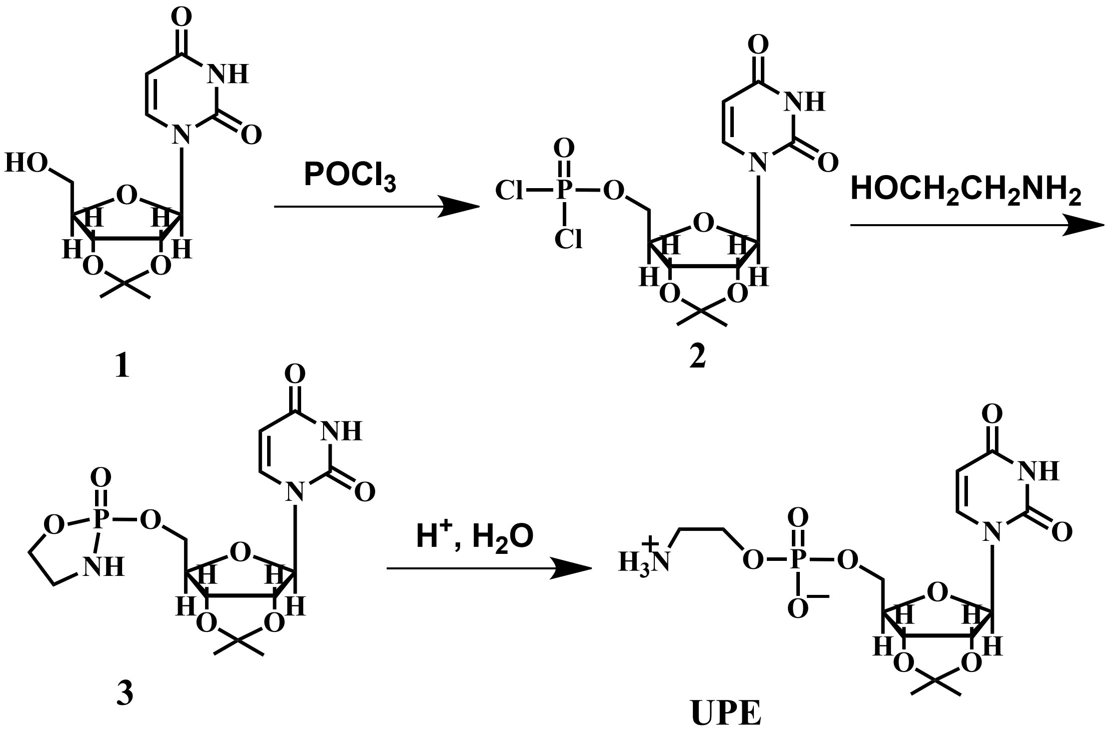

Figure S1. Synthetic route of UPE based on procedure 2.

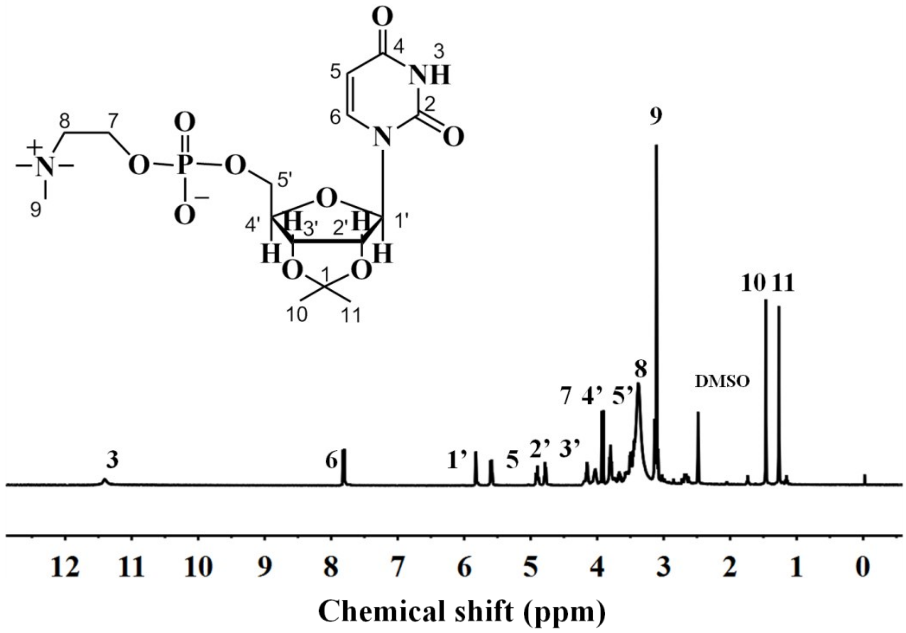

Figure S2. ^{1}H NMR spectrum of UPC in DMSO-d_6.

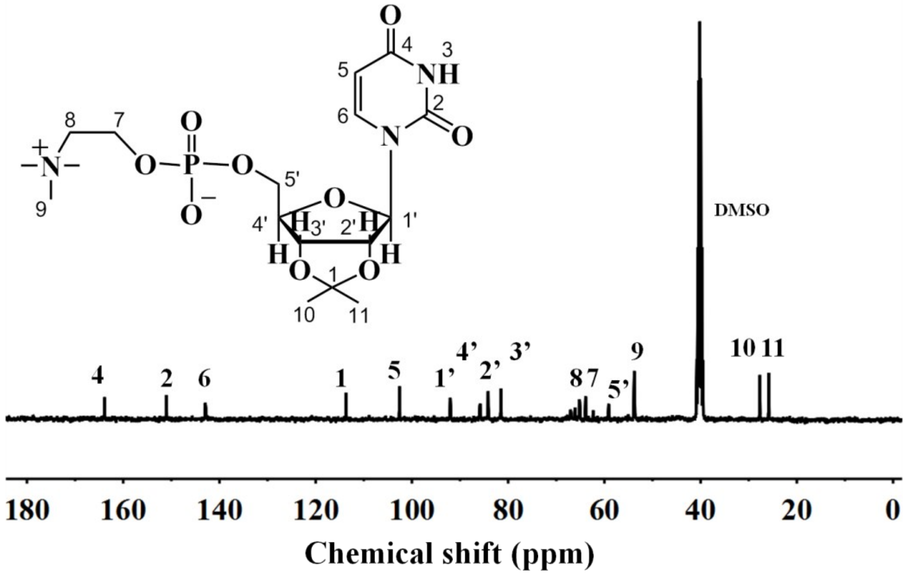

Figure S3. ^{13}C NMR spectrum of UPC in DMSO-d_6.

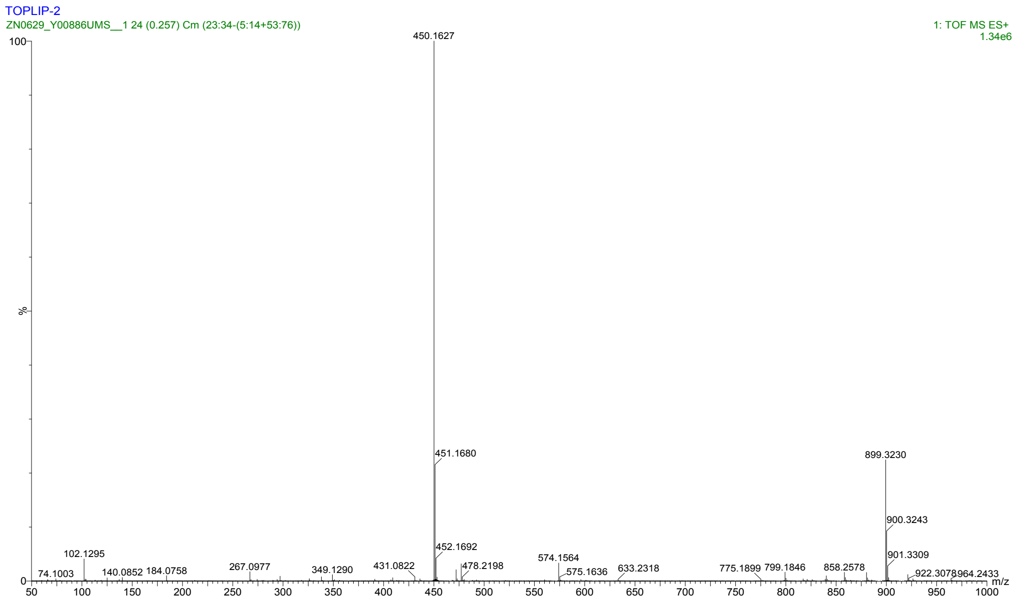

Figure S4. Mass spectrum (ES+) of UPC.

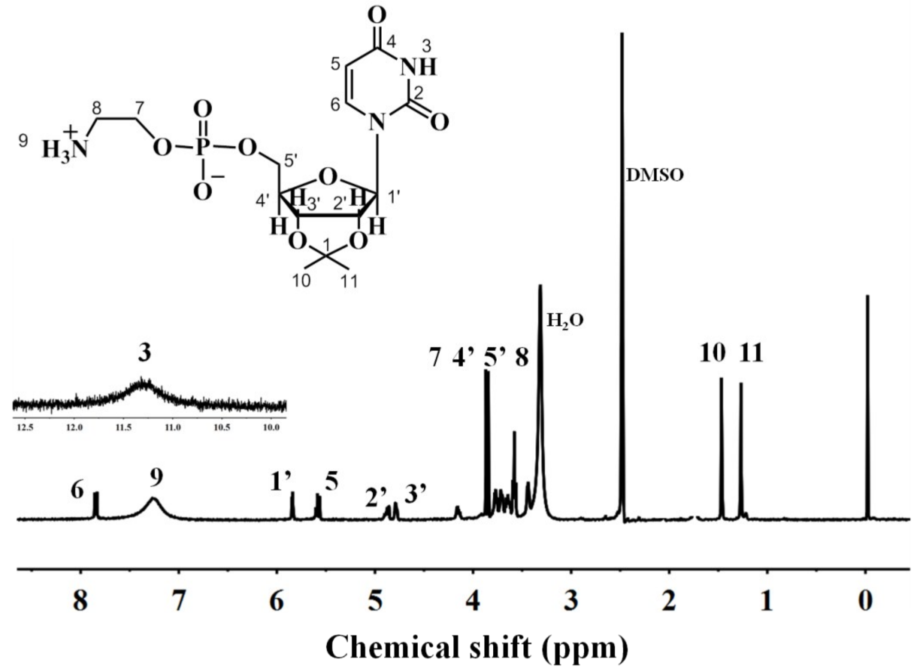

Figure S5. ^{1}H NMR spectrum of UPE in DMSO-d_6.

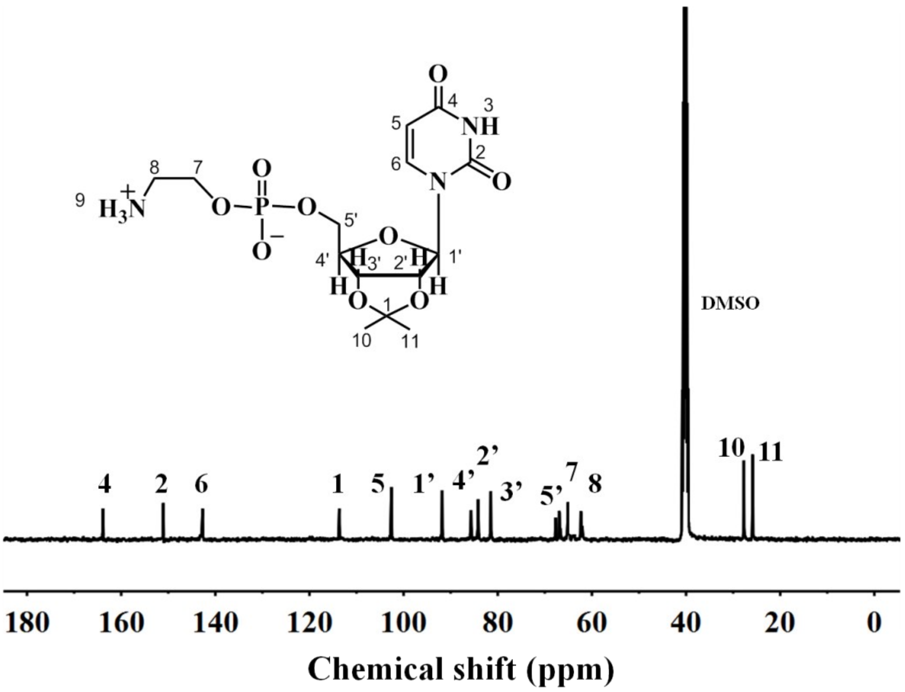

Figure S6. ^{13}C NMR spectrum of UPE in DMSO-d_6.

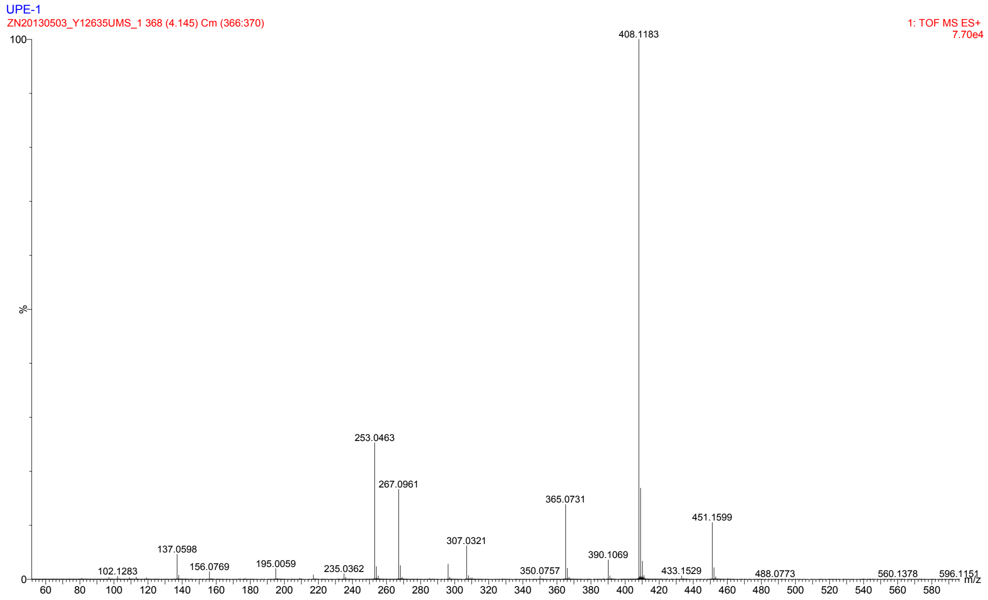

Figure S7. Mass spectrum (ES+) of UPE.

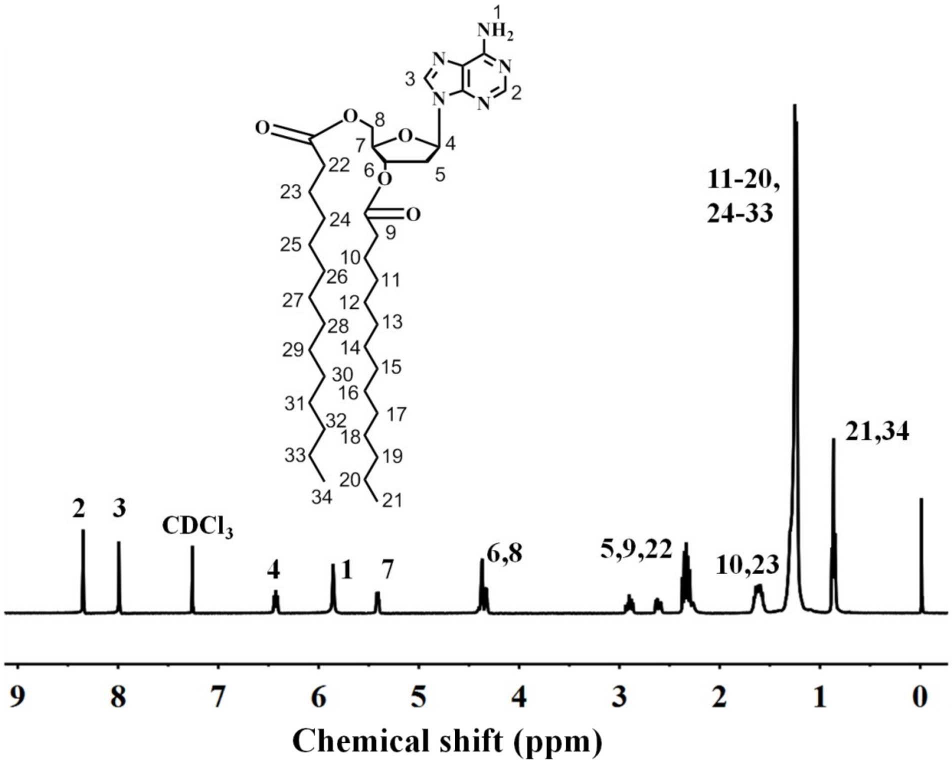

Figure S8. ^{1}H NMR spectrum of DMA in $CDCl_3$.

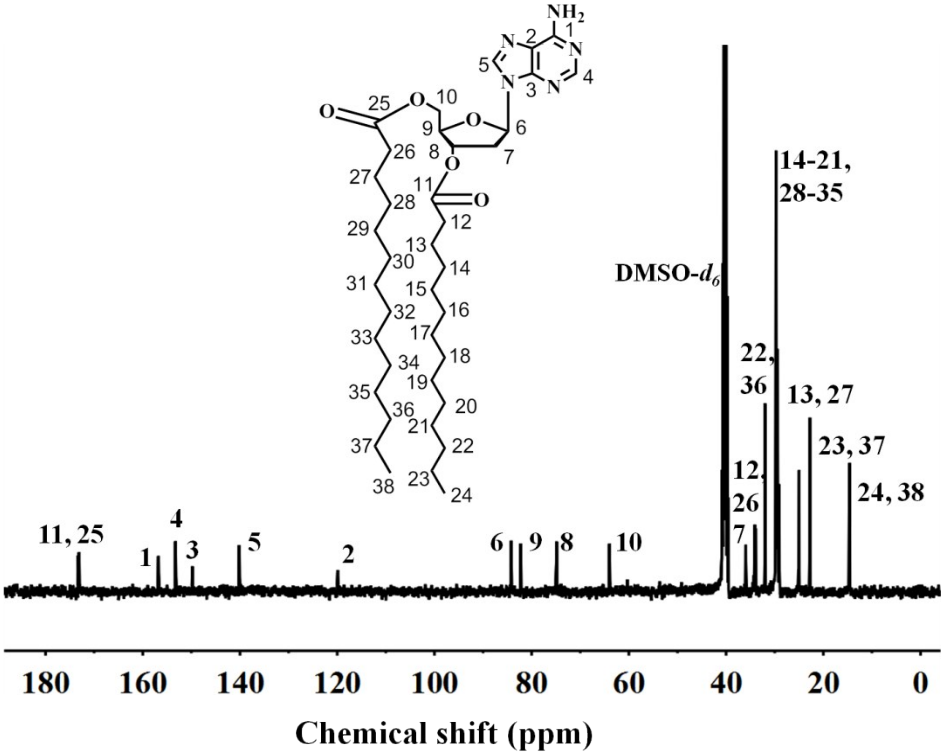

Figure S9. ^{13}C NMR spectrum of DMA in DMSO-d_6.

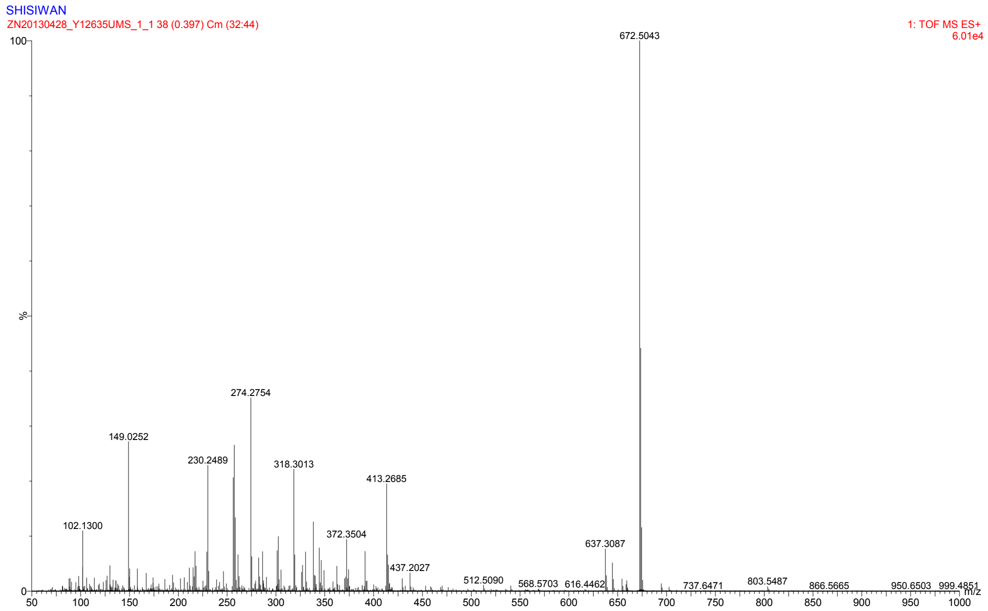

Figure S10. Mass spectrum (ES+) of DMA.

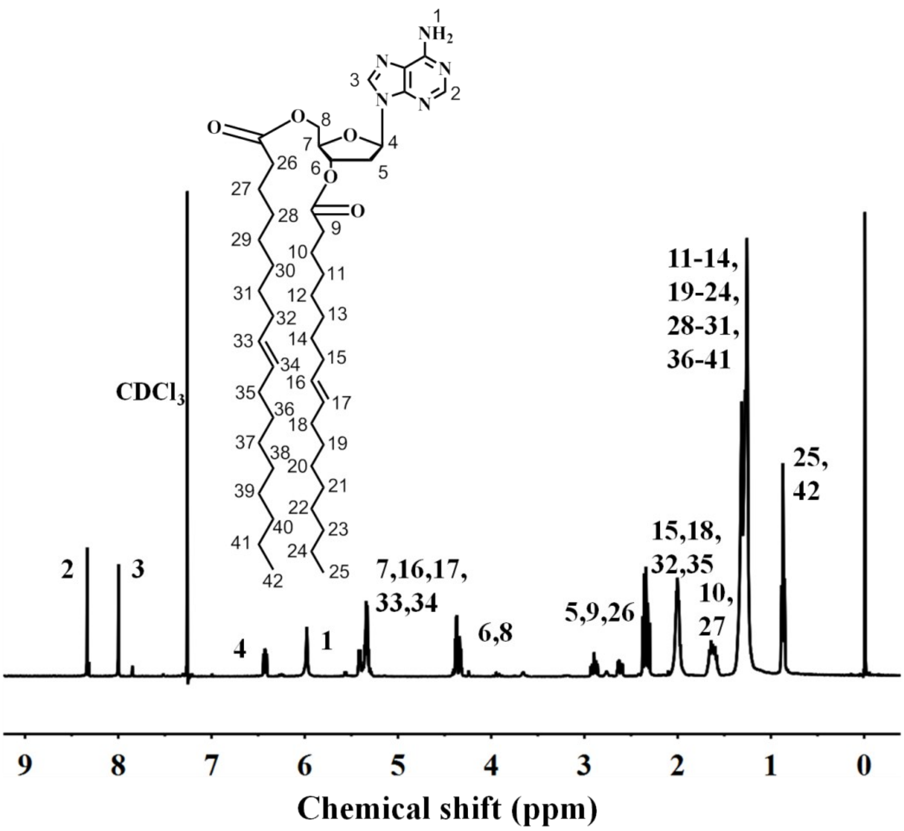

Figure S11. ^{1}H NMR spectrum of DOA in $CDCl_3$.

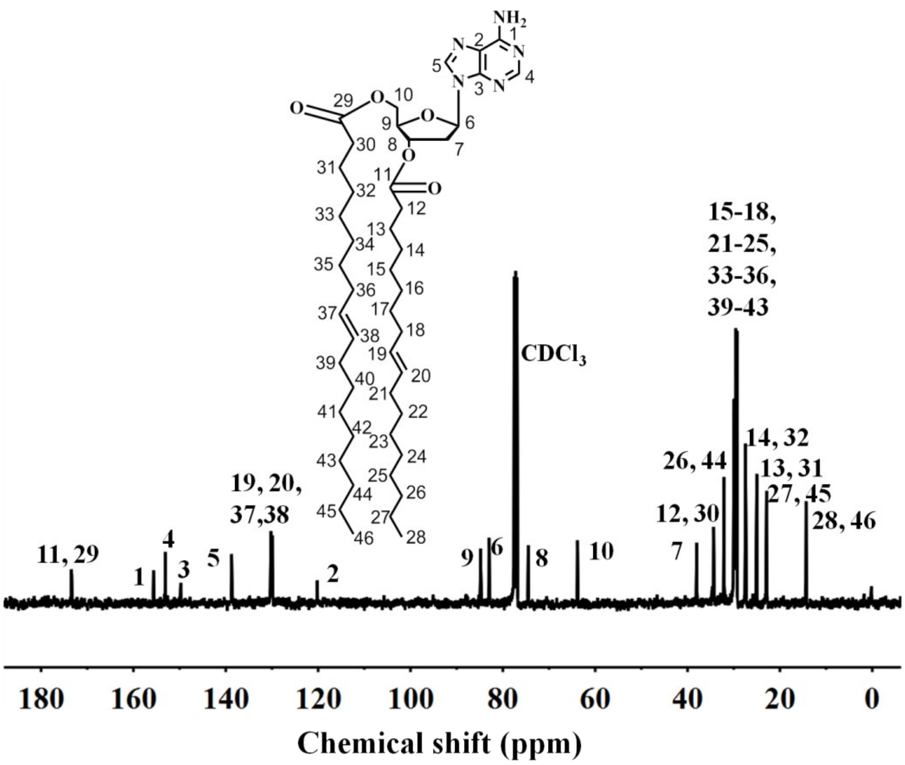

Figure S12. ^{13}C NMR spectrum of DOA in $CDCl_3$.

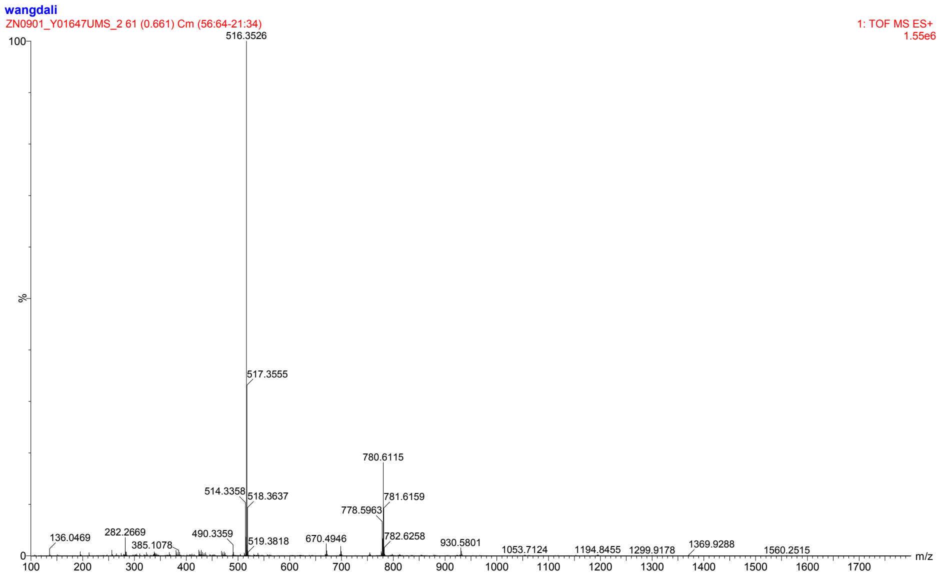

Figure S13. Mass spectrum (ES+) of DOA.

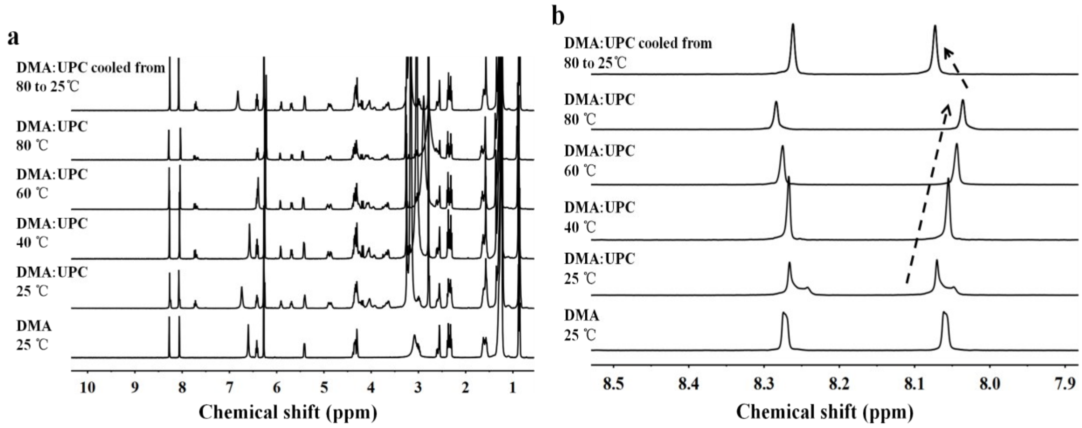

Figure S14. (**a**) Variable temperature ^{1}H NMR spectra of DMA and DMA:UPC. (**b**) Adenine CH chemical shifts of the DMA and DMA:UPC. The mixing ratio (DMA:UPC) was 1:1. Sample was allowed to equilibrate for 5 min at each temperature (1,1,2,2-tetrachloroethane-d_2/dimethylsulfoxide-d_6 = 4/1).

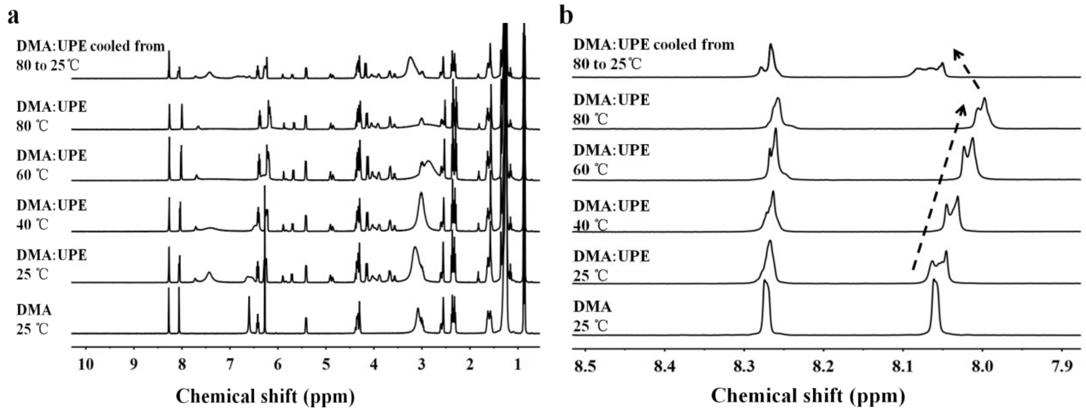

Figure S15. (**a**) Variable temperature ^{1}H NMR spectra of DMA and DMA:UPE. (**b**) Adenine CH chemical shifts of the DMA and DMA:UPE. The mixing ratio (DMA:UPE) was 1:1. Sample was allowed to equilibrate for 5 min at each temperature (1,1,2,2-tetrachloroethane-d_2/dimethylsulfoxide-d_6 = 4/1).

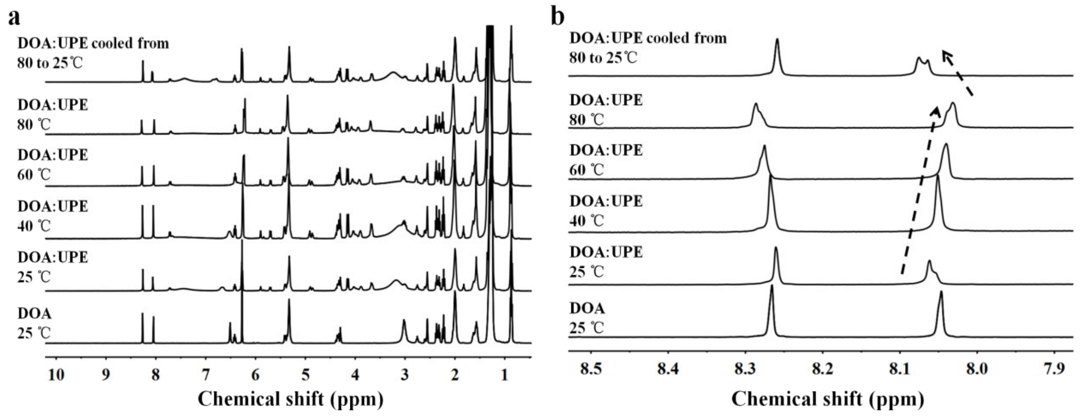

Figure S16. (**a**) Variable temperature ^{1}H NMR spectra of DOA and DOA:UPE. (**b**) Adenine CH chemical shifts of the DOA and DOA:UPE. The mixing ratio (DOA:UPE) was 1:1. Sample was allowed to equilibrate for 5 min at each temperature (1,1,2,2-tetrachloroethane-d_2/dimethylsulfoxide-d_6 = 4/1).

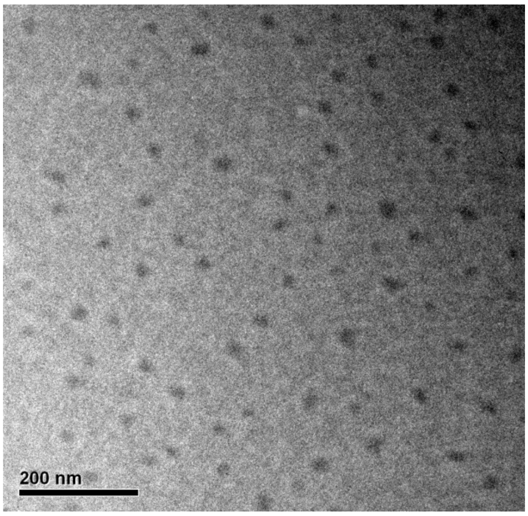

Figure S17. Representative TEM image of micelles self-assembled from pure DOA.

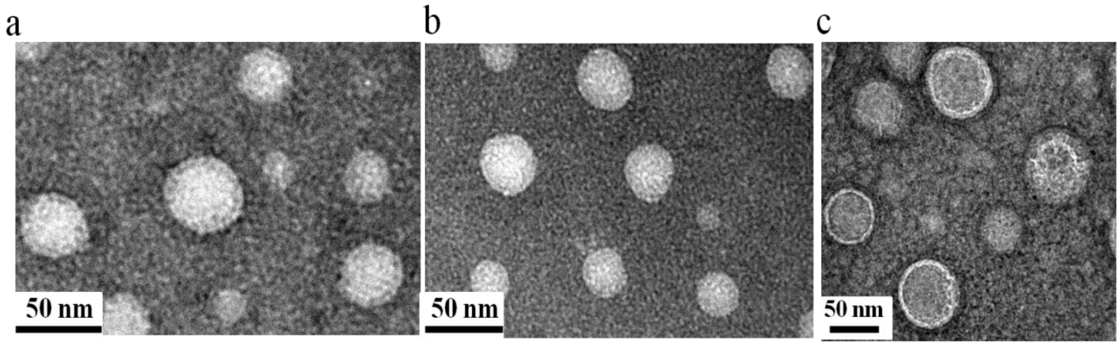

Figure S18. (**a**) Representative TEM image of negatively stained supramolecular DMA:UPC liposomes. (**b**) Representative TEM image of negatively stained supramolecular DMA:UPE liposomes. (**c**) Representative TEM image of negatively stained supramolecular DOA:UPE liposomes.

a

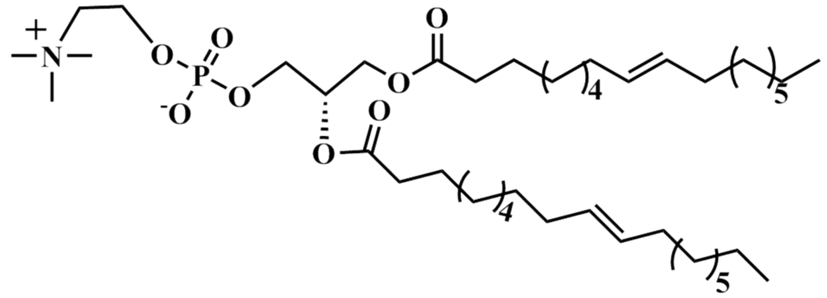

b

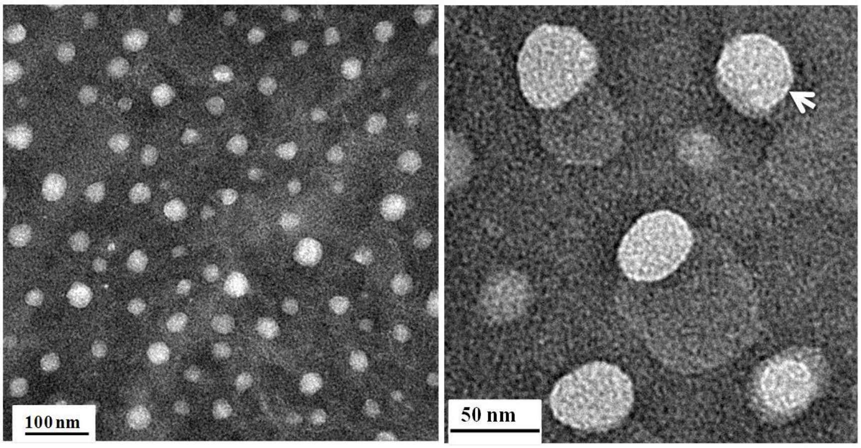

Figure S19. (**a**) The molecular structure of DOPC and (**b**) electron micrographs of negatively stained DOPC-formed conventional liposomes.

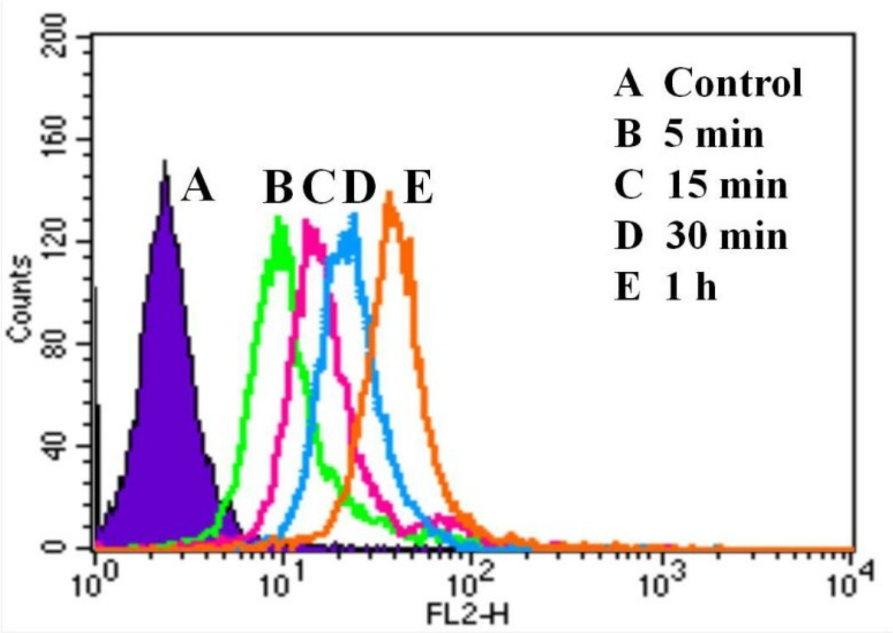

Figure S20. Flow cytometric profiles of MCF-7 cells incubated with DOX-loaded supramolecular DOA:UPC liposomes for different time intervals.

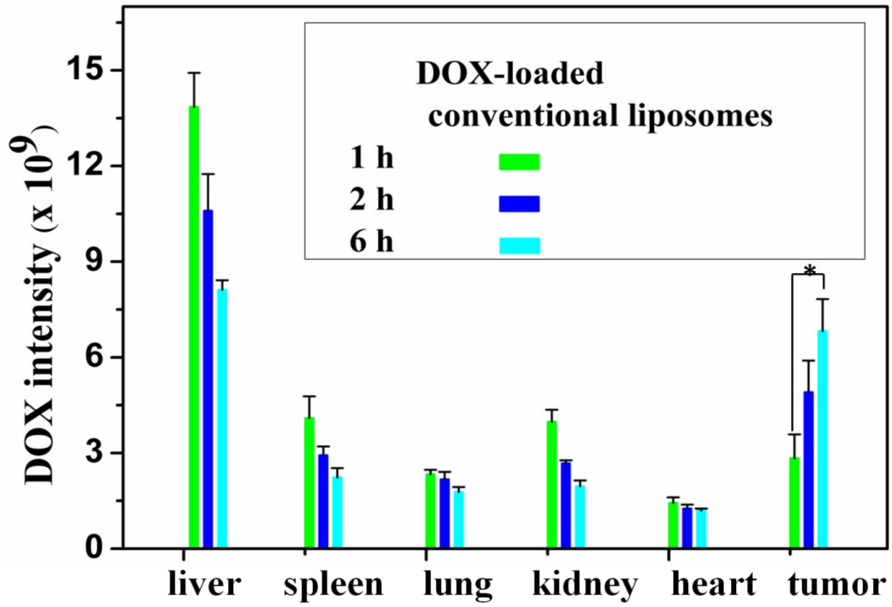

Figure S21. *In vivo* biodistribution of DOX-loaded conventional liposomes administrated intravenous injection to mice. Data are presented as average ± standard error (n = 4), and the statistical significance level is *P < 0.05.

5. References

1 B. Goldenbogen, N. Brodersen, A. Gramatica, M. Loew, J. Liebscher, A. Herrmann, H. Egger, B. Budde and A. Arbuzova, *Langmuir*, 2011, **27**, 10820-10829.
